# Short channel effects on electrokinetic energy conversion in solid-state nanopores

**DOI:** 10.1038/srep46661

**Published:** 2017-04-25

**Authors:** Yan Zhang, Yuhui He, Makusu Tsutsui, Xiang Shui Miao, Masateru Taniguchi

**Affiliations:** 1 School of Optical and Electronic Information, Huazhong University of Science and Technology, LuoYu Road, Wuhan 430074, China; 2 The Institute of Scientific and Industrial Research, Osaka University, 8-1 Mihogaoka, Ibaraki, Osaka 567-0047, Japan

## Abstract

The ion selectivity of nanopores due to the wall surface charges is capable of inducing strong coupling between fluidic and ionic motion within the system. This interaction opens up the prospect of operating nanopores as nanoscale devices for electrokinetic energy conversion. However, the very short channel lengths make the ionic movement and fluidics inside the pore to be substantially affected by the ion depletion/accumulation around the pore ends. Based on three-dimensional electrokinetic modeling and simulation, we present a systematic theoretical study of nanopore electrical resistance, fluidic impedance, and streaming conductance. Our results show that by utilizing the short channel effect and preparing slippery nanopores the energy conversion efficiency can be dramatically increased to about 9% under large salt concentrations.

Nanopores/nanochannels, with their nanoscale radii comparable to the Debye length of electrolytes, are capable of expelling co-ions from outside while attracting counter-ions to the inside due to the surface charges on the channel walls. Such ion selectivity of nanopores/nanochannels has stimulated tremendous research interests because of both the novel transport phenomena at nanoscale and their widespread application potential^1^,^2^,^3^,^4^,^5^,^6^,^7^,^8^,^9^. As demonstrated in Fig. 1a, by imposing a voltage through the pore/channel a longitudinal electro-osmotic flow is induced by the excessive counterions within the pore. In this way the electrical energy is converted into kinetic one. On the other hand, a mechanical pressure-driven flow through the nanopore/nanochannel will carry a net charge within the electrical double layer, inducing an electrical current/potential and thus transforming the energy from mechanical to electric^4^. The maneuverable nanoscale ionic and fluidic transport, and the associated energy conversion can find promising applications such as ion diodes^5^,^10^,^11^, ionic transistors^2^,^7^,^12^,^13^, power-generators^3^,^4^,^14^, water desalination devices^15^,^16^,^17^,^18^ and genome sequencing devices^19^,^20^,^21^. For application purposes, higher conversion efficiency between electrical and mechanical energy is being pursued. Recent experiments showed that a 3% conversion efficiency could be reached in a 75 nm wide nanochannel under very small salt concentrations (*C*_0_ ~ 0.3 mM)^4^. Yet, for feasible applications, this result calls for new strategies to further increase the efficiency, especially at high salt concentrations for the purpose of yielding large power output.

One key difference between nanopores and nanochannels is the geometric aspect ratio, defined as pore/channel length over the radius. For nanopores the lengths are typically on the same nanoscale order of magnitude as the radii, while those of nanochannels are usually in the micrometer scale, which is several orders of magnitude larger than that of the radii. Therefore, the resulting distinction is whether the inhomogeneity of quantities around the pore/channel ends is negligible compared to the more uniform distribution deep inside the pore/channel. For nanochannel systems, the inhomogeneity of ion concentrations and other related quantities at the ends of the channel can be treated as a marginal effect since the length scale of the nonuniform distribution is rather small compared to that of the channel (*L* ~ 10 *μ*m). Based on this approximation, researchers have derived a simplified 1-dimensional (1-D) model by assuming that the nanochannel radial electrostatics can be decoupled from the axial one^1^,^4^,^22^,^23^,^24^. However, inside very short nanopores the ion concentration is *not* invariant along the pore axis due to the influence of the edge effects^25^.

In this paper we show, using electrokinetic modeling and simulations, that the negative charges on the pore wall lead to depletion of Cl^−^ ions while enhancing the K^+^ concentration inside the pore (see Fig. 1b,c). Upon an imposed longitudinal electrical potential difference, there will be depletion of anions around the pore end next to the *trans* chamber (anode side) and their accumulation at the other end (cathode side) (Detail discussion of the physical mechanism will be presented in the following sections). Such polarization of salt concentration along the axial direction was found in ion-selective nanochannel systems^26^. By visualizing both the concentration and electrokinetic flow patten with fluorescently-labeled anionic molecules, Kim *et al*. have observed that there exists an ion-depletion region around the anode side of the channel end, while ion-accumulation area around the other side^26^. The polarization was theoretically predicted to exist even in nanopores where no surface charges were present on the pore wall^25^. In other words, there exists substantial coupling between the axial and radial electrostatics and transport which cannot be evaluated by a simple 1-D model. The question then arises if this coupling enhances or reduces the electrokinetic energy conversion efficiency in nanopore systems. In this work, we present a systematic study on the coupled ionic and fluidic transport in the wall-charged nanopore systems. Based on the illustrated physical mechanisms we will suggest strategies for enhancing/optimizing the energy conversion efficiency.

We start from a full-dimensional description of transport through the nanopore, including Poisson equation for electrostatics, Navier-Stokes equation for laminar flow, and Nernst-Plank equation for ionic motion (See Method section). Then, the nanopore electrokinetic properties are quantitatively characterized by the response of the ionic current *I* and the fluid volume flow rate *Q* to the applied voltage difference *U* or pressure gradient Δ*p*^3^,^27^,^28^,^29^


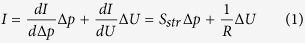



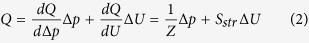


In the above expressions, *Q* is the flow rate defined as 
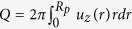
 where *u*_*z*_ is the axial component of fluidic velocity 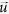 and *R*_*p*_ is the radius of the nanopore; *R* defines the electrical resistance of nanopores; *Z* is the fluidic impedance; *S*_*str*_ is the streaming conductance. According to Onsager relation, *S*_*str*_ = *dQ*/*dU* = *dI*/*d*Δ*p*^30,31^. The figure-of-merit which directly measures the energy conversion efficiency is then defined as follows^3^,^27^,^30^,^31^:


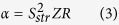


In the above definitions linear relations between flow rate/ionic current and the applied voltage/pressure are assumed. We will discuss the validity of these linear approximations and the associated physical mechanisms. In the following sections, we are going to evaluate the nanopore electrical resistance *R*, fluidic impedance *Z* and streaming conductance *S*_*str*_, paying particular attention to the impact of concentration inhomogeneity from the ends of the nanopore, namely the short channel effect. Finally, we will explore the modulation of transport properties by using slippery pore wall and evaluate the resulting enhancement of energy conversion efficiency.

## Results

### Nanopore electrical resistance *R*

**Figure 2:**
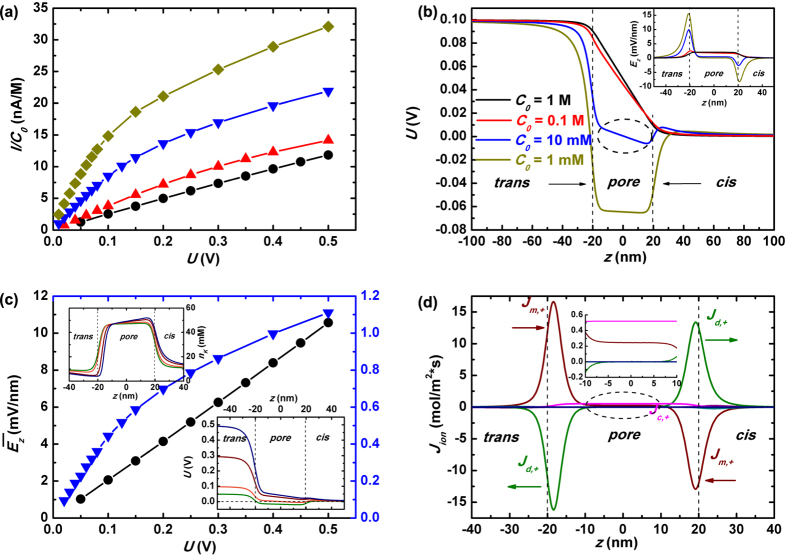
Nanopore electrical resistance. (**a**) The cross-pore ionic current over the imposed salt concentration *I*/*C*_0_ versus the applied voltage *U* under various salt concentrations in a nanopore with radius *R*_*p*_ = 5 nm and length *L*_*p*_ = 40 nm. The black line with round symbols stands for *C*_0_ = 1 M, red line with up triangles for *C*_0_ = 100 mM, blue line with down triangles for *C*_0_ = 10 mM, and dark-yellow line with rhombus symbols for *C*_0_ = 1 mM. This characterization holds for all of the following figures. (**b**) The distribution of the voltage and z-component electrical field *E*_*z*_ (inset) along *z*_0_ under various salt concentrations when a cross-pore voltage *U* = 0.1 V is imposed. (**c**) The averaged E-field 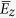 at the pore center (*z* = 0) as a function of the applied voltage *U* under various salt concentrations. The upper-left and lower-right insets plot respectively the distribution of the cationic concentration *n*_*K*_ and voltage *U* along *z*_0_ when the applied voltage *U* increases from 0.05 V (olive line) to 0.1 V (orange line) to 0.3 V (wine line) to 0.5 V (navy line) given the imposed salt concentration *C*_0_ = 10 mM. (**d**) The z-component electrophoretic fluxes *J*_*m*,±_, diffusion fluxes *J*_*d*,±_ and convection fluxes *J*_*c*,±_ of the cations and anions along *z*_0_ where the imposed salt concentration *C*_0_ = 1 mM and voltage *U* = 0.1 V. The wine, olive and pink lines stand for the cationic flux of electrophoresis *J*_*m*,+_, diffusion *J*_*d*,+_ and convection *J*_*c*,+_ respectively, while the violet, dark-cyan and navy lines are for anionic counterparts *J*_*m*,−_, *J*_*d*,−_ and *J*_*c*,−_.

Figure 2a plots our calculation results of ionic current versus applied voltage under various salt concentrations (see Method section). Within the demonstrated voltage range (*U* ≤ 0.5 V) there are several interesting features on the *I(V*) curves: (1) When KCl salt with very large concentration (*C*_0_ = 1 M) is imposed, the ionic current shows a globally linear increasing behavior with the applied voltage. (2) Otherwise, *I(V*) shows two-sectional linearity with a turning point. Below the turning point, a smaller electrical resistance is observed while above it the resistance becomes larger. The smaller the concentration of salt imposed, the more obvious the difference between the two slopes segregated by the turning point.

The above ion transport behaviors do have been reported by the experiments^26^, where a nonlinear *I(V*) characteristic was observed in a perm-selectivity nanochannnel. In order to elucidate the underlying physical mechanisms, we start our investigation from the distribution of pore-axial voltage *U(z*) and electrical driving field *E*_*z*_(*z*). Figure 2b gives a view of the longitudinal voltage dragging down by the negative charges on nanopore wall surface. It further shows that the smaller the salt concentration the stronger the extent of voltage pulling down, which seems rather reasonable considering the poorer screening capability of ions in the solution (Poorer screening capability refers to the fact that under lower salt concentration a larger region within the pore would be affected by the pore-wall surface charges due to the thicker electrical double layers). Yet the above picture of axial voltage pulling down by negative surface charges on pore wall is not an exact explanation, since trapping of voltage along longitudinal direction results in an axial E-field *E*_*z*_ which cannot respond straightforwardly to the pore-wall charges in the radial direction.

The actual physical mechanism can be elucidated from Fig. 2d where various components of axial ionic flux *J*_*z*_ are plotted. Due to the concentration enhancement of cations within the nanopore, there will be significant diffusive cationic flux from the internal parts of the pore to the outside ones. This is marked by the arrows and magnitudes of *J*_*d*,+_ around the pore ends. In order to keep the conservation of total flux along the pore axial direction, the electrophoretic component of cation flux, *J*_*m*,+_, should be of similar magnitude as that of the diffusion while pointing to the opposite direction. That is, *J*_*m*,+_ has to orient from the *trans* chamber (anode side)/*cis* chamber (cathode side) into the nanopore at the pore ends. The above fluxes are distinctly manifested by the associated curves and arrows in Fig. 2d. Such a conservation requirement is fulfilled by the landscape of E-field *E*_*z*_ along the pore axis as shown in the inset of Fig. 2b: it is large in magnitude around the two ends of the pore, while positive at the *trans* chamber side and negative at the other side. This is the physical origin why the electrical field-driven cation flux gets directed in the opposite direction at the two ends. The consequence is that the longitudinal voltage gets dragged down around the pore exit (next to the anode side) and then pulled up when approaching the entrance (next to the cathode side) to shape such a driving field. Moreover, the larger the imposed salt concentration, the better the capability of shielding wall surface charges by the ions within the solution. It leads to a smaller concentration difference between the inside and outside of the pore, and thus yields a smaller diffusive cation flow around the pore ends. This can be observed by comparing the magnitudes of *n*_*K*_ and *J*_*d*,+_ under various sanity concentrations as shown in Fig. S1 of Supplementary material. Consequently, the required axial E-field for balancing the diffusive flow is also reduced under higher salt concentration. The above physical mechanism explains why upon higher salt concentration the axial distribution of voltage *U(z*) is more similar to a linear drop inside the nanopore, while for lower concentration *U(z*) is significantly pulled down inside the pore.

Under very large concentration of KCl (*C*_0_ = 1 M represented by the black line), the longitudinal ionic current *I* shows a globally linear increase with the applied voltage *U*. The rationale behind this has been described above as the excellent capability of screening wall surface charges. Quantitatively, the Debye length is about 0.3 nm when *C*_0_ = 1 M, which leads to extremely thin electrical double layers adherent to the pore wall. Thereby inside the nanopore the axial dropping of voltage *U(z*) and the corresponding E-field *E*_*z*_(*z*) are quite similar to the situation in those pores without wall surface charges (from now on, we define the latter as clean nanopore). This is demonstrated by the black line in Fig. 2b and the black line with round symbols in Fig. 2c.

By contrast, the piecewise linear increase behavior of *I(U*) under smaller salt concentration is a direct consequence of the short channel effect. As shown in the upper-left inset of Fig. 2c, excessive cations are induced by the wall surface charges and are piled up inside the pore. When the applied longitudinal voltage *U* is small (*U* < 0.15 V), the translational invariance of ion distribution along the pore axis ∂*n*/∂*z* = 0 holds valid deep inside the pore (−*L*_*p*_/4 ≤ *z* ≤ *L*_*p*_/4); however, when *U* is larger than a critical value (in our case it is about 0.15 V) the axial distribution of cations *n*_*K*_(*z*) becomes more and more distorted (from orange line to navy line). The cations are being pushed away from the anode-side pore end to the cathode-side one by an increasing force. Such behavior is the response of mobile charges to the imposed cross-pore E-field. In this manner the longitudinal electrostatics and the radial one are coupled together, and translational invariance of ion distribution is broken under a large applied voltage. In response to the decrease of cation concentration at the pore end next to the *trans* chamber and concomitant increase at the other end, the magnitude of electric driving field *E*_*z*_ has to be raised at the former site while reduced at the latter so that the total cation flux remains conserved along the pore axial direction, 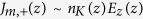. The above short channel effect and conservation requirements explain why more of the imposed longitudinal voltage has to drop around the former site while less at the latter upon larger imposed voltage, as seen in the lower-right inset of Fig. 2c. The reduced E-field finally leads to a lower efficiency of ionic motion response to the imposed large cross-pore voltage, and thus a larger electrical resistance of the nanopore. The phenomenon demonstrated above is quite different from the nanochannel situation since here the short channel effect plays a crucial role in nanopores.

We point out that the above illustration of the ion density polarization can also be used to interpret the hysteresis phenomenon of nanopore ionic current during the voltage scanning^25^. In our nanopore ionic transport experiments, we frequently observe the electrical current hysteresis when reversing the applied voltage. Besides, the lower the imposed salt concentration, the larger the magnitude of the hysteresis. Such facts suggest the existence of charging/decharging process upon the turning upside down of the applied voltage, and the larger amount of the stored charges when smaller concentration of salt concentration is imposed^25^. From the above discussion, we are aware that the ion density depletion/accumulation at the pore ends would result in the storage of net charges, and the amount of these charges would be increased under low salt concentration (An illustration of the net charge density under various salt concentrations is provided in Fig. S1 of the Supplementary Materials). Therefore, the experimentally measured ionic current hysteresis and its dependence on the salt concentration can be attributed to the ion density polarization in the nanopore systems.

### Nanopore fluidic impedance *Z*

**Figure 3:**
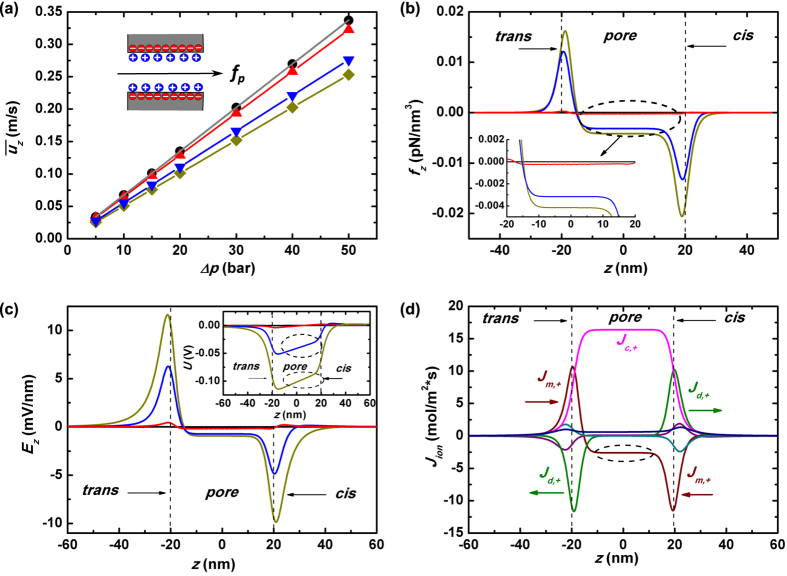
Nanopore fluidic impedance. (**a**) The averaged fluidic velocity 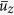 as a function of the applied mechanical pressure Δ*p* under various salt concentrations. The gray line stands for the case of a clean nanopore. Inset gives a schematic view of the pressure-driven fluidics through the nanopore. The induced E-field *E*_*z*_ (**c**) and the z-component electrical body force on the solvent *f*_*z*_ (**b**) along *z*_0_ under various salt concentrations. Inset of (**c**) plots the induced voltage *U* along *z*_0_. (**d**) The z-component electrophoretic fluxes *J*_*m*,±_, diffusion fluxes *J*_*d*,±_ and convection fluxes *J*_*c*,±_ of cations and anions along *z*_0_ given the imposed salt concentration *C*_0_ = 10 mM and pressure Δ*P* = 30 bar. The wine, olive and pink lines are for cationic fluxes of the electrophoresis *J*_*m*,+_, diffusion *J*_*d*,+_ and convection *J*_*c*,+_ respectively, while the violet, dark-cyan and navy lines for anionic counterparts *J*_*m*,−_, *J*_*d*,−_ and *J*_*c*,−_.

Now we turn to the discussion of pressure-driven hydrodynamic flow in the nanopore system. As illustrated in previous sections, the larger the salt concentration *C*_0_, the better its capability of screening the wall surface charges. Thus we expect that larger *C*_0_ should lead to a cross-pore fluidics more similar to the case of clean nanopore. This is sketched in the inset of Fig. 3a, and is demonstrated quantitatively in Fig. 3a where the averaged liquid flow velocity 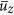 is plotted as a function of the imposed cross-pore hydrodynamic pressure Δ*p*. Here, 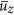 is defined as 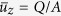, *Q* is the flow rate and 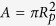 is the cross-section area of the nanopore. The gray line plots 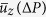 of clean nanopore system, and this line almost overlaps with that where 1 M KCl is imposed in the charged nanopore system.

From the same plot we also notice that the smaller the imposed salt concentration, the lower the averaged flow velocity compared to that of clean nanopores (from red line to blue line to dark-yellow line in Fig. 3a). In other words, smaller salt concentration leads to stronger attenuation of the hydrodynamic flow. Our calculation indicates that poorer capability of shielding pore-wall surface charges causes a loss of the efficiency of pressure-driven flow. Such phenomenon was poorly studied due to theoretical and experimental challenges. In this work, we show a thorough analysis of the short channel effect on the fluidic impedance profiles.

In order to understand the underlying physical mechanism, we go back to the Navier-Stokes equation which depicts force-prompted fluidics as seen in Eq. 9 and Eq. 10. Compared with the fluidics in a clean nanopore, there exists an additional force 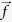 in charged pores due to the imbalanced cations and anion quantities within the solution. We plot in Fig. 3b the calculated z-component force *f*_*z*_ along the pore axis under various salt concentrations, and the inset gives a magnified view of *f*_*z*_ inside the pore. This figure demonstrates that inside the nanopore the body force orients conversely to the pressure driving: *f*_*z*_ points from pore mouth to the exit (−*z*) while the applied pressure propels the solvent from *trans* to *cis* chamber (+*z*, and a 2-D illustration of *f*_*z*_(*r, z*) is provided in Fig. S2 of Supplementary material). The question now becomes why the pore-wall surface-charge-induced force 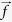 acts as an *opponent* rather than *proponent* to the applied mechanical pressure driving −∇*p*.

From the definition *f*_*z*_ = *ρ*_*e*_*E*_*z*_ and the fact that the excessive ions inside a negatively-charged nanopore are cations (*ρ*_*e*_ > 0), we derive that the z-component E-field should point to the opposite position of pressure-driving. This is verified by our simulation and is shown in Fig. 3c where *E*_*z*_ is plotted along pore axis. The rationale that *E*_*z*_ inside nanopore is oriented to the contrary of pressure driven is going to be interpreted by analyzing Fig. 3d, where various components of cross-pore ionic fluxes are plotted. As demonstrated by the figure, there are electrophoretic fluxes *J*_*m*_, diffusion fluxes *J*_*d*_ and convection ones *J*_*c*_ of both cations and anions. Since the cationic flux overwhelms the anionic one within the pore, we focus our discussion on the former. As shown in the previous section, the directions of *J*_*m*,+_ and *J*_*d*,+_ oppose with each other around the end of the pore. The third component of the cationic flux, namely the convection *J*_*c*,+_, remains almost symmetric at the two ends of the pore, due to the fact that both the fluidic flow and cation concentration are largely symmetric there (Upon no voltage imposed, the distributions of fluid velocity and ion concentration along pore axis are quite symmetric with respect to the pore center, as seen in Fig. S3 of Supplementary material). Then, the law of flow continuity requires the sum of *J*_*m*,+_, *J*_*d*,+_ and *J*_*c*,+_ should remain the same along the pore axis. From the above investigation, we are aware it means


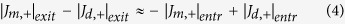


From the nearly symmetric distribution of cations with respect to the pore center along pore axial direction, we derive that the magnitude of diffusive cationic flux *J*_*d*,+_ keeps roughly the same at the two ends: |*J*_*d*,+_|_*exit*_ ≈ |*J*_*d*,+_|_*entr*_. Therefore, it requests that |*J*_*m*,+_|_*exit*_ > |*J*_*m*,−_|_*entr*_ to keep the flux conservation at the two ends. This can be fulfilled by the unequal magnitudes of *E*_*z*_ at the pore entrance and at the exit as |*E*_*z*_|_*exit*_ > |*E*_*z*_|_*entr*_. Such an asymmetric pulling down of the voltage at the two ends of the pore and the resulted larger E-field at the exit are distinctly observed in Fig. 3c: *U* is dragged down much more sharply around the pore exit (*z* ≈ −20 nm) than that at the pore mouth (*z* ≈ 20 nm), and accordingly *E*_*z*_ is much larger at the former location. A direct consequence of this unequal dropping of voltage at the two ends is that *U* has to undergo a moderate increasing deep inside the pore, as marked by the dash circles in the inset of Fig. 3c. This explains why *E*_*z*_ points to the opposite direction of pressure driven.

Figure 3c further indicates that the smaller the salt concentration, the larger the magnitude of the induced E-field. This is once again attributed to the poorer screening of wall surface charges under lower salt concentration. As shown in the inset of Fig. 3c, the cross-pore electrical potential *U* gets more severely pulled down within the nanopore because of those negative charges on the pore wall. Therefore we find that by using smaller salt concentration, not only will the resulted worse shielding capability lead to a substantially reduced electrical driving field under a cross-pore voltage, but it will also cause larger fluidic impedance given an axial pressure driving.

From the above discussion we conclude that the reversed orientation of E-field deep inside the nanopore with respect to that of the hydrodynamical pressure driving is a direct consequence of the pore-wall surface charge dragging. It is this reversed E-field that increases the fluidic impedance of nanopore and consequently, it would enhance the electrokinetic energy conversion efficiency. Our analysis also reveals that such enhancement would be trivial in nanochannel systems where the inhomogeneity of ionic concentrations and fluxes at the channel ends is negligible compared to micrometer-scale uniform distribution of quantities inside.

### Nanopore streaming conductance *S*_*str*_

Eq. 1 and Eq. 2 show that there are two methods of evaluating *S*_*str*_, one by pressure-propelled ionic current, the other by voltage-driven hydrodynamic flow, and results should be the same. In this work we choose the former approach in our simulation since the definition of energy conversion efficient comes directly from the mechanical-to-electric energy transforming^3^.

**Figure 4:**
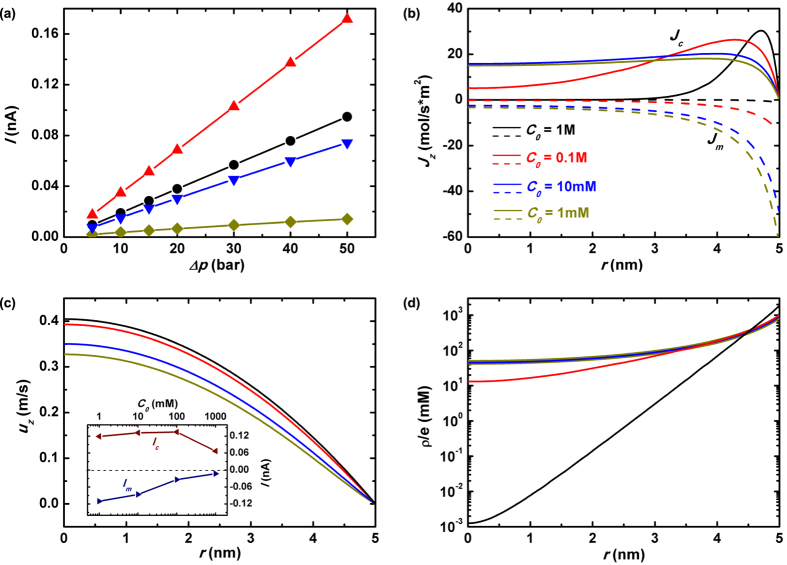
Nanopore streaming conductance. (**a**) The ionic current *I* versus the imposed cross-pore pressure Δ*p* in a *R*_*p*_ = 5 nm and *L*_*p*_ = 40 nm nanopore under various salt concentrations. The pore-radial distribution of the z-component ionic fluxes *J*_*z*_ (**b**), fluidic velocity *u*_*z*_ (**c**) and net charges *ρ*_*e*_/*e* (**d**) at *z* = 0 nm with a longitudinal driving pressure Δ*p* = 30 bar. Besides, in (**b**) the real lines characterize the convection flux *J*_*c*_ while dash lines represent the electrophoretic flux *J*_*m*_ which are defined as *J*_*m(c*)_ = *J*_*m(c*),+_ − *J*_*m(c*),−_. The inset of (**c**) plots the components of electrical current due to the ionic convection *I*_*c*_ and electro-migration *I*_*m*_ as functions of the imposed salt concentrations. The former is marked by the wine line with left-triangle symbols, while the latter by the navy line with right-triangle symbols.

Figure 4a plots the calculated ionic current *I* as a function of the applied mechanical pressure Δ*p* under various salt concentrations *C*_0_. It is intriguing to see that the slope, which is the streaming conductance *S*_*str*_, becomes largest at *C*_0_ = 100 mM, rather than at smallest *C*_0_ = 1 mM or at largest *C*_0_ = 1 M. The illustrated characteristics differs significantly from the prediction by conventional modeling. According to the traditional analysis, the pressure-driven electrical current was estimated through 
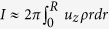
. The full amount of *ρ* in the channel cross-section was determined by the wall surface charge density, as required by charge neutrality condition:


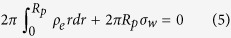


The fluid velocity *u*_*z*_ was then roughly determined by the imposed pressure Δ*p*. Thus the streaming current *I* appeared to be independent on the sanity concentration *C*_0_ at first glance. However, smaller *C*_0_ causes larger proportion of the induced counterions to locate near the radial center of the pore (see Fig. 4d), where the fluid velocity is largest. Therefore, conventional model suggested that *S*_*str*_ kept increasing with the decreased salt concentration and finally became saturated at quite small *C*_0_.

Nonetheless, the above analysis cannot be applied to streaming current in nanopores once again due to the short channel effect. As shown in Fig. 4b, c and d, we plot the pore-radial distribution of ionic convection flux *J*_*c*_(*r*) = *J*_*c*,+_ − *J*_*c*,−_, electrophoretic flux *J*_*m*_(*r*) = *J*_*m*,+_ − *J*_*m*,−_, cross-pore fluid velocity *u*_*z*_(*r*) and the net charge *ρ*/*e* = (*n*_*K*_ − *n*_*Cl*_) at the pore-axial center (*z* = 0) under mechanical pressure driving. The diffusion flux of ions is not shown since deep inside the pore it is negligible. According to our analysis shown in the previous section, the cross-pore fluid velocity *u*_*z*_ is largest under highest salt concentration *C*_0_ = 1 M due to the best charge-shielding capacity. Yet it does not necessarily lead to largest ionic convection current at *C*_0_ = 1 M because of the net charge distribution. As seen in Fig. 4d, when the imposed concentration of KCl is very large most of the net charges are stringently adhered to the pore wall. However, it is the very place where the convection velocity is smallest due to the friction by the pore wall. Therefore the efficiency of transforming mechanical motion to electrical current falls to the lowest when the salt concentration is too high. This is quantitatively demonstrated by comparing *J*_*c*_ under various salt concentrations shown in Fig. 4b. Such a failure on the other hand indicates that by making nanopore wall surface more slippery, a substantially enhanced ionic current can be expected. This is of particular interest from the viewpoint of practical application since output power with orders of magnitude enhancement will be harvested under such situation. Theoretical study of this topic will be deployed in the next section.

The key difference between our simulation and the traditional modeling lies in the prediction of streaming conductance behavior under small salt concentrations. Our results indicate a decreasing trend while the 1-D model displays saturation tendency with reduced *C*_0_. Based on our previous discussion of nanopore electrostatics under mechanical pressure driving (Δ*p* → *E*_*z*_), we are aware that smaller salt concentration will result in larger magnitude of contrarily directed E-field *E*_*z*_. That is why the reversely oriented electro-migration flux of net charges, *J*_*m*_, grows larger under decreased salt concentration as shown by the dash lines in Fig. 4b. On the other hand, neither the radial distribution of net charges *ρ(r*) nor fluid velocity *u*_*z*_(*r*) varies too much under very small *C*_0_ as seen in Fig. 4c,d, which suggests that the convection component of electrical flux *J*_*c*_ will keep roughly the same. This is further demonstrated by our evaluation of convectional and electrophoretic ionic current, *I*_*c*_ and *I*_*m*_, defined as 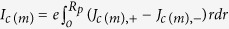 shown in the inset of Fig. 4c. Thereby our conclusion is that under very small salt concentrations, the streaming conductance will be reduced due to the increased electrophoretic flow of ions in the opposite direction. We would like to point out that our conclusion shows qualitative agreement with the experimental observations^4^,^32^.

### Impact of slippery wall

Recent experiments point to situations where hydrodynamic slip occurs at the interface between water and nanochannel surface^33^,^34^,^35^,^36^. A significant enhancement in the efficiency of electrokinetic energy conversion has been predicted theoretically, due to the resulting increase in the streaming conductance^28^,^29^,^37^. Experimentally, water and molecule transport through nanoscale carbon pipes with speeds orders larger than earlier theoretical expectation has been reported^33^,^34^,^38^,^39^,^40^. Besides, giant osmotic energy conversion efficiency has been reported by using boron nitride nanotubes as nanochannels^35^, implying that water flow in such pipes is nearly frictionless. Here we present a theoretical study of transport in slippery nanopore systems, illuminating physical mechanisms and suggesting strategies of promoting the electrokinetic energy conversion efficiency.

For understanding the altered transport behavior, the central issue is the reshaping of pressure and voltage throughout the system when the nanopore turns from non-slip to slippery. At first glance, inside the pore the fluidic motion should be substantially advanced due to the elimination of frictions by the wall, and so is the increasing of the convectional counterion flux along the pore wall. Yet from the viewpoint of flow continuity there should be a simultaneous enhancing of the liquid and ionic flux *outside* the pore to match the changes *inside*. It is particularly worth noticing those changes at the nanopore ends due to short channel effect. Such changes could only be accomplished by the self-adaptive reshaping of cross-pore pressure and voltage: *p(z*) and *U(z*) have to raise the fluidic rates of liquid and ions around the pore ends, while suppress the overly enhancing tendency of them within the pore when the channel surface becomes frictionless. In the following we are going to discuss transport in a slippery nanopore under pressure driving (under voltage driving discussion is provided in Supplementary material).

#### Pressure-driven transport

**Figure 5:**
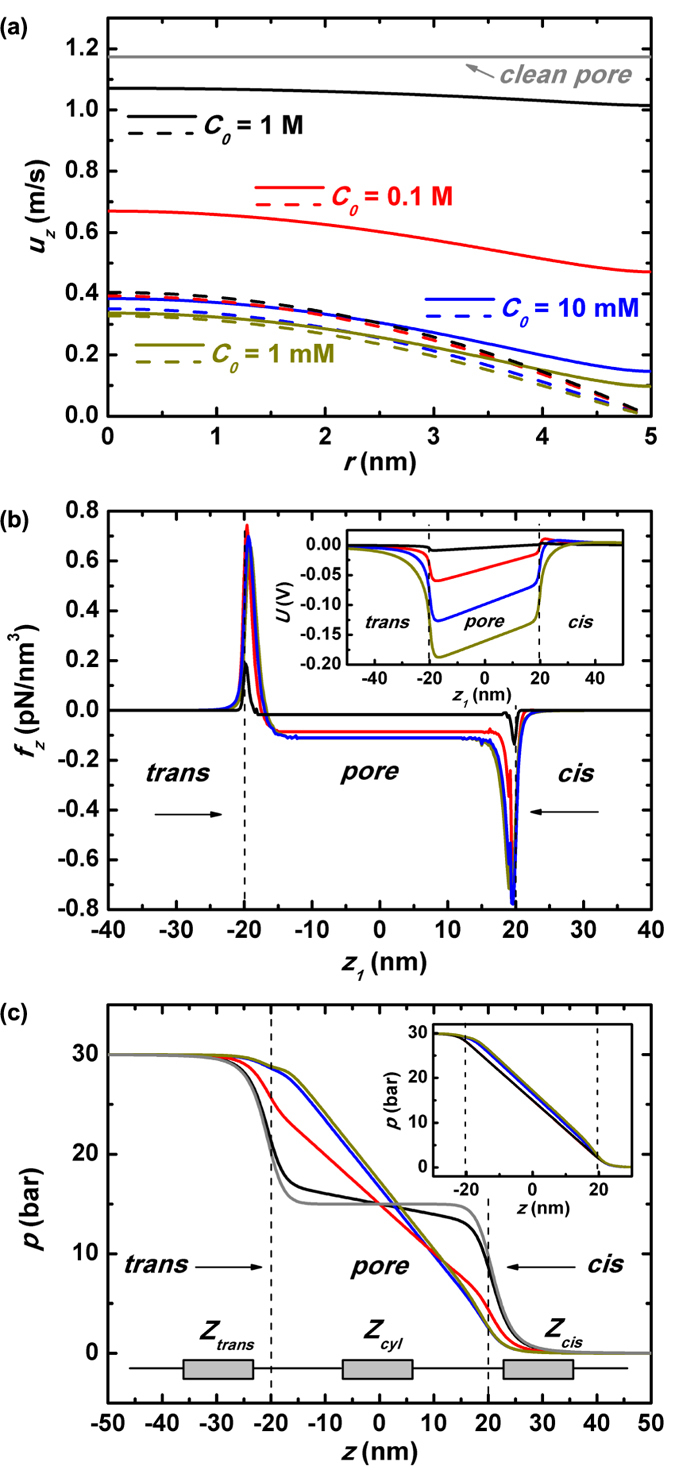
Fluidics in a slippery nanopore. (**a**) The pore-radial distribution of z-component fluidic velocity *u*_*z*_(*r*) at the pore center (*z* = 0) under various salt concentrations while the gray line shows that in a clean nanopore. In the plots, the real lines are for the slippery nanopore while dash lines are for the non-slip situation. (**b**) The distribution of z-component electrical body force on the fluidics *f*_*z*_(*z*_1_) along *z*_1_ axis (*r* = 4.5 nm) as marked in Fig. 1c, and the inset plots the potential distribution along that line *U(z*_1_). Here some post-simulation smoothing has been performed on the curves to eliminate the unphysical fluctuations by finite-element methods. (**c**) The distribution of the applied pressure *p(z*) along *z*_0_ under various salt concentrations, while the inset of (**c**) plots that within a non-slip nanopore. *Z*_*cis*_, *Z*_*trans*_ and *Z*_*cyl*_ denote the fluidic impedance of the two chambers and the cylindrical pore regions.

Figure 5a plots the radial distribution of the cross-pore fluid velocity *u*_*z*_(*r*) at the pore-axial center (*z* = 0) under various salt concentrations. The applied pressure difference Δ*p* is 30 bar. The real lines are for slippery nanopore while the dash lines for non-slip one. It is just as expected that by using frictionless pore the liquid flow is significantly expedited, especially near the channel wall, Yet it further demonstrates that the higher the imposed salt concentration the better the expedition. Does it imply that stronger capability of screening pore-wall surface charges will lead to more unrestrained liquid movement, just like what occurs in a clean nanopore? To answer this question, *u*_*z*_(*r*) within a clean and slippery nanopore is presented by gray line in Fig. 5a. It verifies that under very large salt concentration *C*_0_ = 1 M, the landscape of fluid speed is quite close to the situation inside a clean nanopore. On the other hand, lower salt concentration would lead to smaller enhancement of the fluidic motion and consequently less reduction of fluidic impedance when the pore wall turns from frictional to frictionless. From discussion in previous sections, we are aware that this is attributed to the reversely oriented E-field *E*_*z*_ which is induced to balance the axial ionic current. The magnitude of such a field keeps increasing when the imposed salt concentration decreases and thus exerts larger drawing-back force *f*_*z*_ on the pressure-driven fluid motion. Under slippery boundary condition, the mechanism remains working as show in Fig. 5b where the z-component electrical body force on the liquid *f*_*z*_ is plotted along the pore axial line *z*_1_. The inset of Fig. 5b is the pore-axial variation of electric potential *U*. These plots show a very similar physical picture as that in non-slip nanopores (Fig. 3b,c), but with larger magnitude of voltage dragging-down. It indicates a *resistive* E-field *E*_*z*_ and electrical body force *f*_*z*_ with larger amplitude would be resulted in. We are going to illustrate that the increased parts of *E*_*z*_ and *f*_*z*_ are response to the nonslip-to-slip change of nanopore. As the pore wall changes from viscous to frictionless, both the liquid flow *Q* and convectional cation flux *J*_*c*,+_ should be greatly enhanced. Such a big change could not occur inside the pore locally, and there should be self-adapted tuning of pressure *p* and electric driving field *E*_*z*_ both inside and outside of the pore to match the flows at the two spaces. The pressure is a propelling force while E-field is a resistive one. In principle the former should be allocated more outside the pore so that flows get larger there *J*_*z*_|_*out*_↑, and the latter should be reinforced inside the pore to suppress too large scale of flow enhancement there *J*_*z*_|_*in*_↓. The latter explains why severer pulling down of axial voltage is observed in slippery nanopores.

The pore-axial distribution of the applied pressure *p(z*) is plotted in Fig. 5c to give a more direct view of the fluidic impedance distribution in a frictionless nanopore system. The gray line in the figure characterizes *p(z*) in a clean nanopore where most of the imposed pressure difference Δ*p* drops at the two ends of the pore. This can be understood from the equivalent fluidic circuit shown at the lower corner of Fig. 5c. The three impedors, *Z*_*cis*_, *Z*_*pore*_ and *Z*_*trans*_ representing impedance of *cis* chamber, pore region and *trans* chamber, are in series. For a frictionless and clean nanopore *Z*_*pore*_ would be profoundly reduced and consequently, a much smaller share of Δ*p* would fall inside the pore. Under large salt concentration *C*_0_ = 1 M, the geometric configuration of *p(z*) is quite close to that of clean nanopore due to its superior capability of shielding pore-wall charges. Yet the figure shows that under small salt concentrations, the pressure distribution curves do not change too much comparing to that in a nonslip nanopore (blue and dark-yellow lines in the inset of the figure). In other words, ∂*p*/∂*z* keeps the linear drop within the slippery pore if KCl salt is imposed with small concentration.

**Figure 6:**
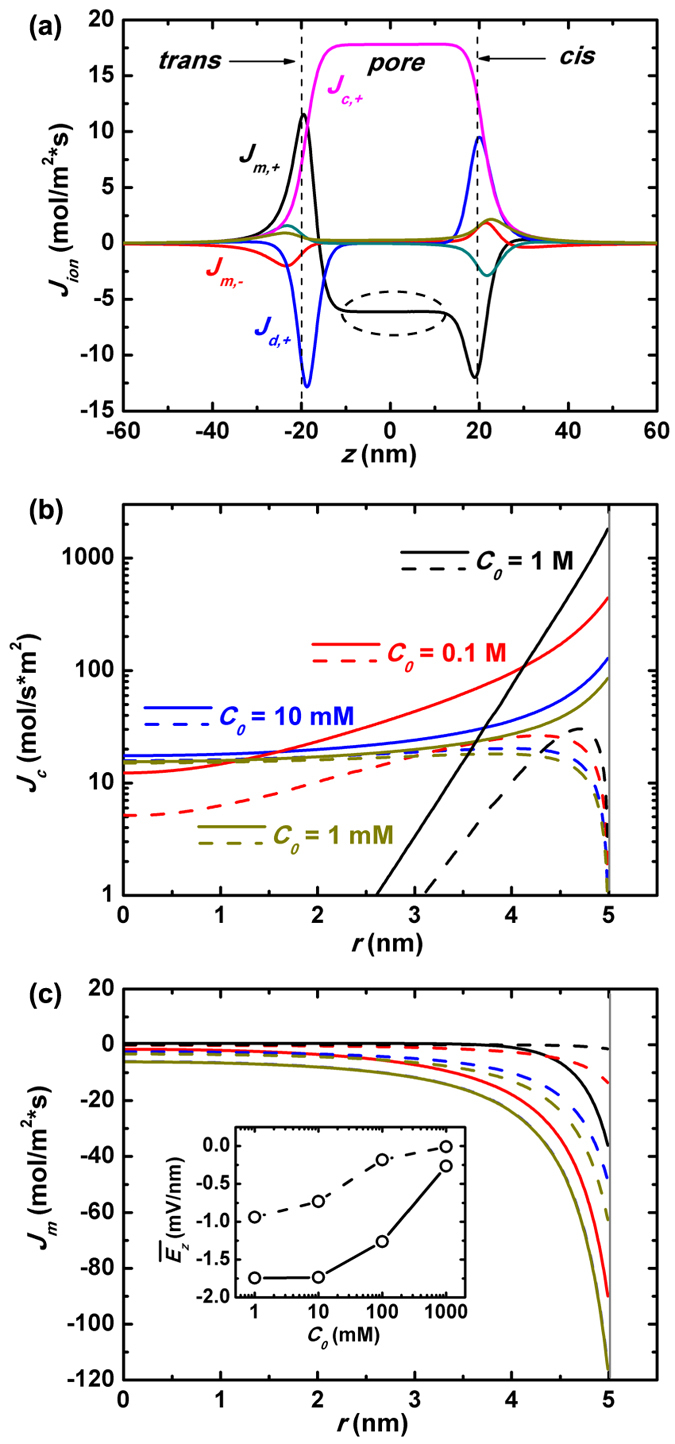
Streaming conductance in a slippery nanopore. (**a**) The distribution of various components of z-component ionic flux *J*_*z*_ along *z*_0_ under salt concentration *C*_0_ = 10 mM. (**b**) and (**c**) The pore-radial distribution of the convection ionic flux *J*_*c*_(*r*) and electrophoretic flux *J*_*m*_(*r*), where the real lines are for the slippery nanopore while the dash lines are for the non-slip nanopore. The inset of (**c**) plots the pore-radial averaged electric driving field 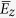 at *z* = 0 as a function of the imposed salt concentration *C*_0_, where the real line with symbols is for the slippery nanopore and the dash line for the non-slip one.

Here the physical mechanism is still the requirement of liquid flow and ionic flux continuity under smaller salt concentration. First we make a distinguishing of the E-field driving capacity between large salt concentration situation and small one. Upon larger *C*_0_, the net charges induced by pore-wall surface charges are more concentrated to the thin layer adherent to the pore, leaving most of the pore space almost uncharged (see black line in Fig. 4d). That is, given the same *E*_*z*_ it will result in much smaller electrical body force *f*_*z*_ on the liquid since *f*_*z*_ = *E*_*z*_*ρ*_*e*_. Therefore, under large imposed *C*_0_ both the profiles of pressure and voltage have to be adjusted when the pore wall becomes frictionless. The former is for the purpose of decreasing driving force on liquid motion inside nanopore, while the latter for slowing cationic movement there. In the contrary, under small *C*_0_ merely the redistribution of *E*_*z*_ itself is sufficient for tuning both fluidic and ionic transport. This is demonstrated by examining Figs 3b,d, 5b and 6a together. Under *C*_0_ = 10 mM, the increasing of *E*_*z*_ will lead to larger amplitudes of both electrical dragging-back force *f*_*z*_ on the liquid and reversely-oriented electrophoretic flux *J*_*m*,+_. The former is seen by comparing blue curves in Figs 3b and 5b, the latter by black curves in Figs 3d and 6a (see the region marked by the dash circles).

From the above discussion, we are aware that the responses to the viscous-to-frictionless change of nanopore wall are quite different under various salt concentrations. When large concentration of salt is added, both the pressure and voltage are re-distributed to match the changes of fluidic and ionic transport. On the other hand, only the distribution of voltage is obviously tuned for the changes under small salt concentration.

**Figure 7:**
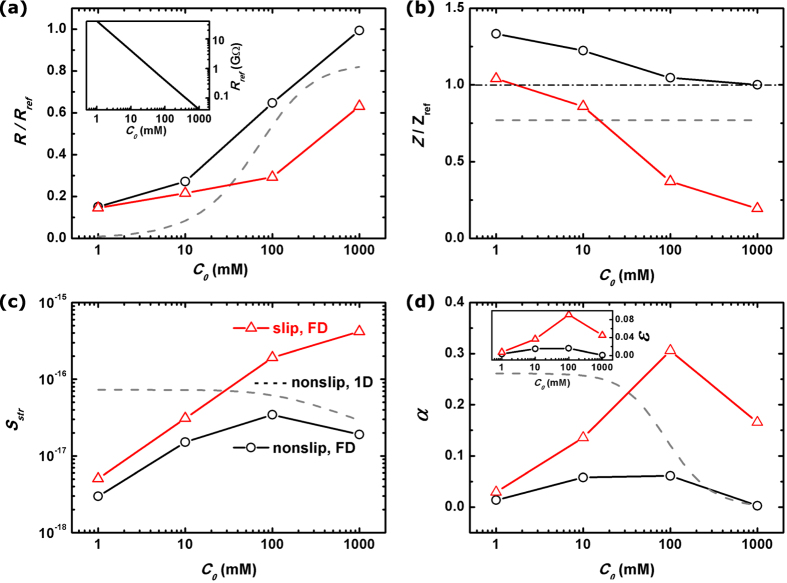
Nanopore figure of merit. The calculated electrical resistance *R* (**a**), fluidic impedance *Z* (**b**), streaming conductance *S*_*str*_ (**c**) and figure of merit *α* (**d**) of a nanopore system with *R*_*p*_ = 5 nm and *L*_*p*_ = 40 nm. The solid lines with round symbols stand for our full-dimensional electrokinetic simulation, where the nanopore walls are non-slip, while the red lines with triangle symbols are for the slippery nanopore systems. The gray dash lines are the results by the conventional 1-D modeling of non-slip nanochannels. In (**a**,**c**), the electrical resistances and fluidic impedance of a clean nanopore, *R*_*ref*_ and *Z*_*ref*_ are taken as references. *R*_*ref*_ of a 10 nm-diameter and 40 nm-long nanopore is further plotted as a function of *C*_0_ in the inset of (**a**). The energy conversion efficiencies *ε* for slippery/non-slip nanopores are plotted in the inset of (**d**).

The radial distribution of convectional and electrophoretic fluxes of net charges upon the pressure driving, *J*_*c*_(*r*) and *J*_*m*_(*r*), are plotted in Fig. 6b,c (The simulation results of *J*_*c*_(*r*) based on experiment data^35^ are plotted in Fig. S7 of Supplementary material). The magnitudes of them under various salt concentrations are as expected based on the above analysis. It suggests that in a slippery nanopore, the pressure-driven ionic current will be greatly improved when large concentration of salt is added. The correspondingly enhanced streaming conductance *S*_*str*_ is of particular interest from the viewpoint of application, since it points to large output power. The shapes of 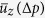 and *I*(Δ*p*) curves of slippery nanopore systems are quite similar to those of non-slip counterparts shown in Figs 3a and 4a, except with different slope values. We put the calculated *Z* and *S*_*str*_ in the final discussion (Fig. 7) for comparison while leave 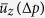 and *I*(Δ*p*) curves in Fig. S5 of Supplementary material.

### Figure of merit

Figure 7 sums up the major results in this work: the variation trends of nanopore resistance *R*, fluidic impedance *Z*, streaming conductance *S*_*str*_ and figure of merit *α* with the salt concentration *C*_0_ are demonstrated for both non-slip nanopore systems (real black lines with symbols) and the slippery ones (real red lines with symbols). For a clear illustration that to what extent the presence of pore-wall surface charges modulate the electric response of nanopore system, we take the resistance of a clean pore as a reference in Fig. 7a:


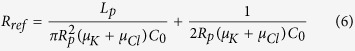


The figure shows a significant decrease of the resistance when the nanopore is charged on the wall surfaces. Besides, the smaller the imposed salt concentration, the larger the ratio of decrease. From previous discussion we are aware that these are caused by the excessive cations in the channel. These counterions are induced by the pore-wall surface charges and then make the additional contribution to the electrical conduction. The amount of such induced charges *ρ*_*e*_ is actually determined by the pore-wall surface charge density *σ*_*w*_ due to the electrical neutrality requirement Eq. 5. Therefore the smaller the imposed salt concentration, the larger the proportion of amounts of those *extra* contributors to the *ordinary* ones, which is roughly estimated as 2*σ*_*w*_/*eC*_0_*R*_*p*_. In other words, 
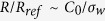
 when *C*_0_ is small. That explains the observed relation between *R*/*R*_*ref*_ and *C*_0_ in the figure (the gray line). Moreover, when *C*_0_ becomes very small, *R*/*R*_*ref*_ seems to stop decreasing and become saturate. The physical mechanism was explained in the previous section as that under smaller salt concentration the electrical driving field decreases substantially due to the short channel effect. Finally, the red line shows that by introducing slippery pore wall the electrical resistance is further decreased and such a decreasing is more obvious at large salt concentrations. We attribute this to the fact that near the channel surface the fluid motion receives the largest improvement when using frictionless wall, and most of the induced charges that are concentrated in this water layer thereby attain a substantially enhanced convection movement.

For discussing nanopore fluidic properties, we take the simulated impedance of a clean and nonslip nanopore as a reference *Z*_*ref*_. Figure 7b shows that the surface charges on the pore wall will result in larger impedance than the reference, and the smaller the salt concentration the larger the promotion of *Z*. As elucidated previously, this impedance enhancement is ascribed to the induced electric field pointing to the opposite direction of pressure driving. Such a *resistive* field is developed for the purpose of preserving continuity of pore-axial counterion flow. It has to be larger to balance the increased diffusive counterion flux at the pore ends when smaller concentration of salt is imposed. The by-product is that a stronger dragging-back electrical body force is exerted on the fluid, therefore leading to larger impedance. Furthermore, when the channel inside the pore becomes slippery the fluidic impedance submits to a great reduction. As seen by red line in Fig. 7b such a reduction reaches maximum under high salt concentration. The related rationale is that the excellent capacity of screening pore-wall charges by high concentration of ions would lead to more similar fluidic behavior as that in a clean and frictionless nanopore.

The streaming conductance *S*_*str*_ characterizing the turning from mechanical flow to electrical current is plotted in Fig. 7c. It shows that in a nonslip nanopore neither too large nor too small concentration of salt will achieve the highest conversion efficiency. The former situation is rendered by the fact that under large sanity concentration most of the conductive net charges are piled up in the extremely thin layer adhered to the channel wall, where the fluidic motion is just slowest due to friction from the wall. Thus the convectional flux of net charges, which contributes most to the induced ionic current, is severely restrained. The latter, on the other hand, is jeopardized by the induced *resistive* E-field which orients to the reversed direction of convectional cation flux under small salt concentration. As shown previously it is a short channel effect of nanopore that cannot be described properly by conventional 1-D model (gray line). Then, by utilizing nanopores with frictionless channels the streaming conductance will be improved by 5~22 times under large salt concentrations as we compare quantities on the red and black lines of Fig. 7c. Such promotion of streaming conductance becomes largest when the imposed KCl concentration is highest (*C*_0_ = 1 M). The phenomenon is once again ascribed to the fact that the enhancing of convectional current of net charges, Δ*I*_*c*_, would be of largest magnitude when the induced cations are concentrated in the thin layer adjacent to the pore wall and thus benefit the most from the friction-to-frictionless turning of channel surface.

At first glance, the promotion of *S*_*str*_ by slippery channel seems quite inspiring for the prospect of high efficiency and large output power application. However, this enhancement is attenuated by two factors: a decrease in electrical resistance which promotes dissipation, and a reduction in fluidic impedance which reduces the available mechanical energy for conversion. The figure of merit *α*, that directly measures the efficiency of mechanical-to-electrical energy conversion in outer circuit, is then plotted in Fig. 7d. According to traditional 1-D modeling of nanochannel transport, in nonslip nanochannel system *α* should keep increasing with decreased salt concentration and finally become saturated at sufficiently low concentration (C_0_ ~ 0.1 mM for their device dimensions)^3^,^28^. Here we demonstrate a very different physical picture that *α* would be optimized at some moder ate salt concentration, which we ascribe to the short channel effect. The maximum coefficient of electrokinetic energy conversion^3^,^4^, defined as 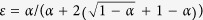, is further plotted in the inset of Fig. 7d. It indicates that for our slippery nanopore system, a conversion efficiency of 9% can be reached at *C*_0_ = 100 mM. It is the best trade-off between streaming current and electrical dissipation when the imposed concentration of salt is about that value. Values of *R, Z, S*_*str*_ and *α* under various salt concentrations in both nonslip and slippery nanopore systems are provided in the tables of Supplementary material.

Moreover, double-cone pore structure has been observed in the experiments^41^, we have also discussed the influence of round corner of the nanopore on transport properties for interested readers (see Supplementary material).

## Discussion

We have shown that a full-dimensional electrokinetic modeling and simulation are essential for evaluating the electrokinetic transport in nanopore systems. The physical mechanism is that the distribution of cation and anion concentrations and other quantities are far from uniform along the pore axis due to the influence of ion concentration polarization at the pore ends. Such short channel effect makes the transport in nanopore systems remarkably different from that in the nanochannels according to our theoretical analysis. We have investigated systematically the electrical resistance, fluidic impedance and streaming conductance, which are the three crucial parameters for characterizing the efficiency of electrokinetic energy conversion in nanopores. Our results have demonstrated that under lower salt concentrations the curves of electrical current versus applied voltage would show two-sectional linearity rather than proportional relation globally. Besides, the fluidic impedance of nanopore systems under the pressure driving would be promoted if the imposed concentration of salt is small. The streaming conductance will be optimized at some moderate salt concentration, rather than monotonous decreasing or saturation behavior. The revealed novel transport phenomena have been traced back to the physical origin of short channel effect in the nanopore systems, and detailed illustration of the associated physical pictures has been presented in the work. Based on the simulation and analysis, we have concluded that the electrokinetic energy conversion efficiency will be optimized at some moderate salt concentrations and will be affected by the edge shape of the nanopore. Furthermore, we have predicted that by using slippery pore wall it will boost substantially the energy conversion efficiency at large salt concentrations to about 9%, which is of particular interest for the pursuit of large power output by the research community.

## Methods

2-dimensional z-axis symmetric model calculating ion transport, laminar flow and electrostatics along z-direction and nanopore radial direction was built up with COMSOL4.4: (1) Poisson equation for electrostatics


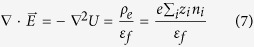


In the above equation, 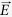 is the electric field, *U* is the electrical potential, *ρ*_*e*_ is the net charge density, *ε*_*f*_ is permittivity of the fluid, *n*_*i*_ is the concentration of the *i*^*th*^ ionic species, and *z*_*i*_ is the valency. Besides, on the pore wall, we applied the boundary condition of surface charges as


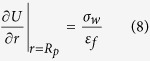


where *σ*_*w*_ is the surface charge density on the pore wall. The boundary conditions for the nanopore and chamber walls are electro-neutrality 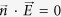. When an electrical voltage *V*_0_ is imposed, the boundary conditions at the ends of the chambers are *U*|_*z*=∞_ = *V*_0_ and *U*|_*z*=−∞_ = 0. Otherwise, upon only hydrodynamic pressure Δ*p* is imposed, the electrical neutrality boundary conditions are used instead.

(2) Steady-state Navier-Stokes equation for liquid motion


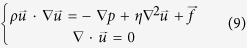


where 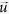 is the velocity of the liquid, *p* is the hydrostatic pressure, *η* is the fluid viscosity and 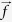 is the body force exerted on the fluid. In our study, 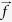 is the electrostatic force due to excessive cations induced by the negative charges on the pore wall:


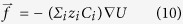


The boundary conditions for the nanopore and chamber walls are no-flux and non-slip: 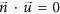 and 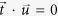. When a mechanical pressure Δ*p* is imposed, the boundary conditions at the two ends of the chambers are *p*|_*z*=−∞_ = Δ*p* and *p*|_*z*=∞_ = 0.

For non-slip wall we assume 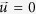 at the pore wall *r* = *R*_*p*_, while for slip wall we apply the boundary condition:


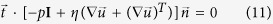


where 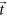 and 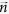 are the tangential and normal directions along the pore wall surface. In our nanopore system, it becomes ∂*u*_*z*_/∂*r* = 0 at the pore wall.

(3) Steady-state Nernst-Plank equation for ion motion


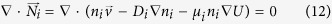



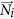
 is the ionic flux density of the *i*^*th*^ ionic species, *D*_*i*_ is the diffusivity, and *μ*_*i*_ is the mobility.

Parameters used in the calculation: mobility of K^+^ and Cl^−^: *μ*_*K*_ = 7.616 × 10^−8^ m^2^/sV and *μ*_*Cl*_ = 7.909 × 10^−8^ m^2^/sV; solution permittivity *ε*_*f*_ = 7.08 × 10^−10^ F/m; fluid viscosity *η* = 8.91 × 10^−4^ Pa ⋅ S. The nanopore is assumed to be made by SiO_2_ and thus the surface charge density *σ*_*w*_ = −49 mC/m^2^ 1,23. The nanopore is assumed to be made by SiO_2_ and thus the surface charge density *σ*_*w*_ = −49 mC/m^2^ 1,23. As discussed in the previous section, whether the channel or pore short depends on the geometric aspect ratio, defined as pore/channel length over the radius. The smaller the geometric aspect ratio, the more obvious short channel effect. So we choose the pore geometry *R*_*p*_ = 5 nm and *L*_*p*_ = 40 nm in this paper to fully study the short channel effect of nanopore.

Supporting Figures for discussing transport in a *R*_*p*_ = 5 nm and *L*_*p*_ = 40 nm nanopore are presented, and the values of *R, Z, S*_*str*_ and *α* under various salt concentrations in both nonslip and slippery nanopore systems are provided in the tables. Streaming conductance calculated from voltage-driven fluid is discussed.

## Additional Information

**How to cite this article:** Zhang, Y. *et al*. Short channel effects on electrokinetic energy conversion in solid-state nanopores. *Sci. Rep.* **7**, 46661; doi: 10.1038/srep46661 (2017).

**Publisher's note:** Springer Nature remains neutral with regard to jurisdictional claims in published maps and institutional affiliations.

## Supplementary Material

Supplementary Information

## Figures and Tables

**Figure 1:**
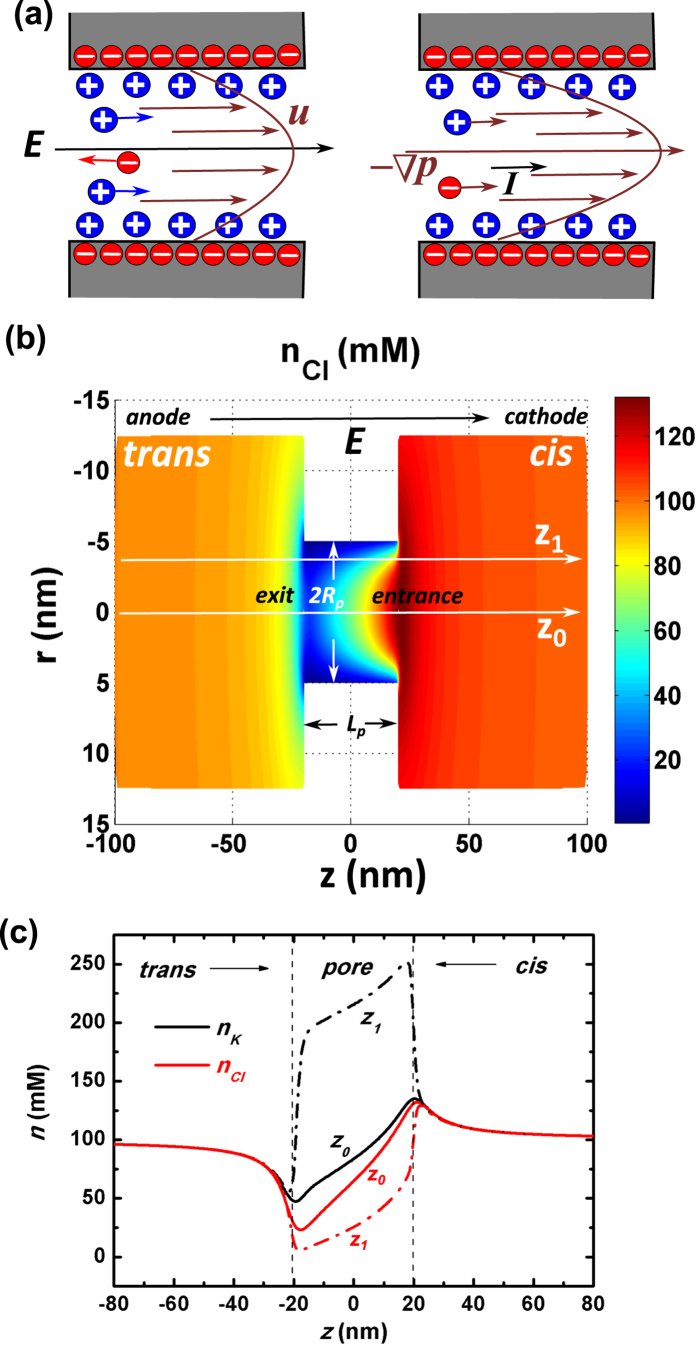
(**a**) A schematic view of the electrical voltage-driving and mechanical pressure-driving fluidic and ionic transport through a nanopore system where negative charges are presented on the pore wall. (**b**) The simulated 2-dimensional radial-axial (*r* − *z*) distribution of Cl^−^ in a nanopore system *n*_*Cl*_(*r, z*) when a cross-pore voltage *U* = 0.3 V is applied. Here the nanopore radius *R*_*p*_ = 5 nm, the pore length *L*_*p*_ = 40 nm, and the imposed salt concentration *C*_0_ = 100 mM. *z*_0_ characterizes the pore axis, while *z*_1_ is a line within one Debye length from the pore wall. (**c**) The concentrations of K^+^ *n*_*K*_ (black lines) and Cl^−^ *n*_*Cl*_ (red lines) along *z*_0_ (real lines) and *z*_1_ (dash-dot lines).
