# Supplementary material for: Short channel effects on electrokinetic energy conversion in solid-state nanopores
Source: Sci Rep. 2017 Apr 25;7:46661. doi: 10.1038/srep46661 (PMC5404231; doi:10.1038/srep46661)
Supplement: Supplementary Information [file srep46661-s1.pdf]

Supplementary Material for Short channel effects on electrokinetic energy conversion in solid-state nanopores

Yan Zhang[1], Yuhui He[1,+], Makusu Tsutsui[2,*], Xiang Shui Miao[1,*], and Masateru Taniguchi[2]

[1]School of Optical and Electronic Information, Huazhong University of Science and Technology, LuoYu Road, Wuhan 430074, China
[2]The Institute of Scientific and Industrial Research, Osaka University, 8-1 Mihogaoka, Ibaraki, Osaka 567-0047, Japan
[*]corresponding.author.tsutsui@sanken.osaka-u.ac.jp
[+]these authors contributed equally to this work

ABSTRACT

The ion selectivity of nanopores due to the wall surface charges is capable of inducing strong coupling between fluidic and ionic motion within the system. This interaction opens up the prospect of operating nanopores as nanoscale devices for electrokinetic energy conversion. However, the very short channel lengths make the ionic movement and fluidics inside the pore to be substantially affected by the ion depletion/accumulation around the pore ends. Based on three-dimensional electrokinetic modeling and simulation, we present a systematic theoretical study of nanopore electrical resistance, fluidic impedance, and streaming conductance. Our results show that by utilizing the short channel effect and preparing slippery nanopores the energy conversion efficiency can be dramatically increased to about 9% under large salt concentrations.

Voltage-driven transport

The convectional and electrophoretic fluxes of net charges along the pore radial direction, $J_c(r)$ and $J_m(r)$, under longitudinal voltage driving are plotted in Fig. S6a and b. The variation of these fluxes when nanopore turns frictionless is illustrated by comparing the real lines and dash ones where the former represent slippery pore situation while the latter are for the nonslip one. We find that the convectional ionic current is substantially promoted due to the much enhanced speed of fluid next to the slippery wall. The promotion of J_c is particularly prominent at high salt concentrations since many more net charges are concentrated in the thin layer adherent to the pore wall where the fluid motion increases the most. However, the electro-migration current of ions J_m seem to be reduced upon the change of pore-wall surface smoothing property. According to the curves shown in the inset of Fig. S6b, such a reduction is caused by the decreased E-field inside the pore. The radial-averaged E-field at pore-axial center $(z = 0)$ $\bar{E_z}$ before and after the channel surface becomes hydrodynamically frictionless are marked by the dash and real line with symbols respectively. We observe that electrical driving field for cation motion is reduced upon the smoothing change of pore wall surface. The physical mechanisms are to be demonstrated as follows by analyzing the altering of voltage and pressure. Fig. S6c shows the pore-axial distribution of the imposed voltage $U(z)$ while the inset show that of pressure $p(z)$. We can see the scenario under voltage-driving is quite similar to that under pressure driving from the viewpoint of changes of both propelling and resistive forces in response to the frictional-to-frictionless conversion of nanopore wall. The only difference between voltage-driven situation and the pressure-driven one is that currently the imposed voltage/E-field becomes the forwarding force while self-adapted pressure is the dragging-back one. By comparing the shapes of red, blue and dark-yellow lines in this figure and those in Fig.2b of **Main context**, we are aware that deep inside the pore the voltage falls more slowly under smaller salt concentrations when the nanopore becomes slippery (see the regions marked by dash circles in the figures). It results in smaller electric driving field E_z deep inside the pore while larger E_z around the pore ends so that the cationic current is suppressed inside and meanwhile promoted outside. This is the current conservation requirement for the nonslip-to-slip change of nanopore as discussed previously.

On the other hand, given large salt concentration C_0 there is no obvious variation of the voltage distribution as shown by comparing black line in Fig. S6c and that in in Fig.2b of **Main Context**. It suggests that the electrophoretic motion of cations does not vary too much deep inside the pore, which is also reflected by the small difference between the real and dash black lines shown in Fig. S6b. The retarding of total cationic current therefore is accomplished via the reducing of convectional speed of ions as seen in the inset of Fig. S6c. When $C_0 = 1$ M, the axial distribution of pressure undertakes

a significant change which results in a dragging-back hydrodynamic force on the liquid ($-\partial p/\partial z$ ¡0). In this manner, the too-large increasing trends of both cross-pore fluid and cationic convection motion due to the nonslip-to-slip conversion of pore wall get suppressed and the total flow rates remain conserved. The shapes of $I(V)$ curves for a slippery nanopore system under various salt concentrations are quite similar to those of non-slip counterparts shown in Fig.2a of **Main context**. We put the calculated R in the final summing-up Fig.8 of **Main context** while leave $I(V)$ curves in Fig. S5.

The influence of round corner of the nanopore on transport properties

Besides, we have also discussed the influence of the round edge of the nanopore (the radius is defined as R_{corner} shown in the Fig. S8) which has been observed in experiments[1] As shown in the results, the deviation (it means the deviation of the calculated nanopore resistance R, impedance Z and streaming conductance S_{str} in nanopore with R_{corner} from that without considering R_{corner}.) increases with larger R_{corner}, while the overall curve trend are consistent with the main context. Interestingly, the nanopore energy conversion efficiency will be enhanced with larger R_{corner} (Fig. S12). The physical mechanism is that although the nanopore electric resistance R (Fig. S9), fluidic impedance Z (Fig. S10) will decrease with increasing R_{corner} and the streaming conductance (Fig. S11) will keep increasing. Eq.3 in the main context indicates that the figure of merit depends on the square of S_{str}. Therefore, the enhancement of S_{str} compensates the reduction of R and Z, with increasing round corner radius. The above discussion may provide useful suggestions for nanopore theoretical and experimental researchers. For resistance deviation, we define

$$D = \frac{R_{R_{corner}} - R_0}{R_0} \tag{S1}$$

For fluid impedance deviation, we define

$$D = \frac{Z_{R_{corner}} - Z_0}{Z_0} \tag{S2}$$

For streaming conductance, we define

$$D = \frac{S_{R_{corner}} - S_0}{S_0} \tag{S3}$$

References

1. Kim, M. J., Wanunu, M., Bell, D. C. & Meller, A. Rapid fabrication of uniformly sized nanopores and nanopore arrays for parallel dna analysis. *Advanced materials* **18**, 3149–3153 (2006).

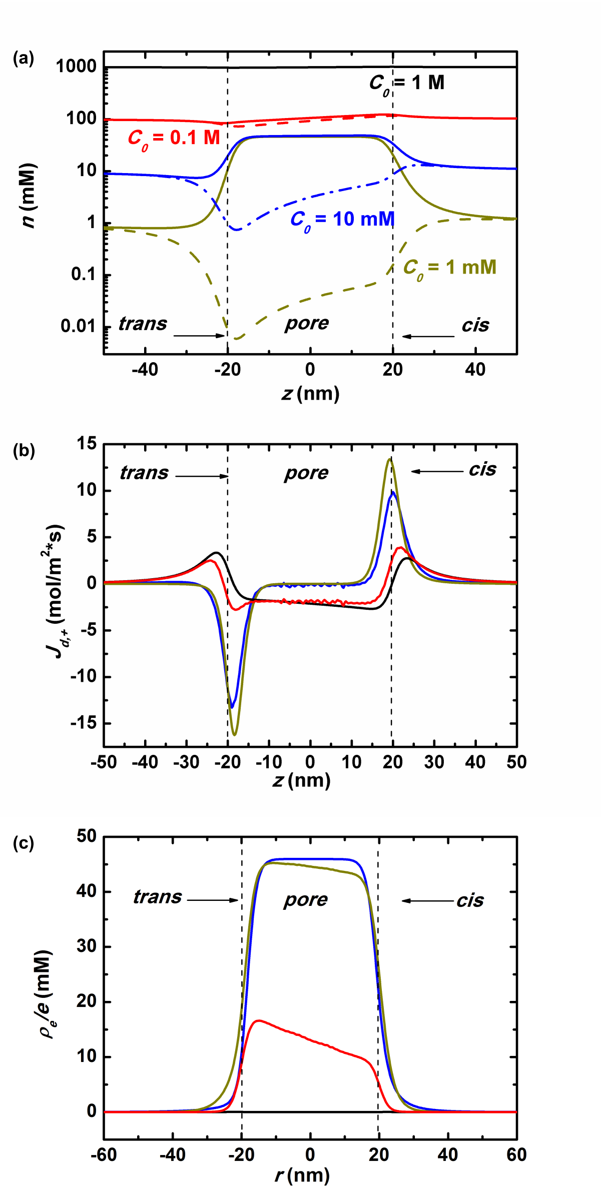

Figure S1. The pore-axial distribution of cation and anion concentrations, $n_K(z)$ and $n_{Cl}(z)$ (a), z-component diffusion cationic flux $j_{d,+}(z)$ (b), and the net charge density ρ_e/e. The real lines and dash lines represent $n_K(z)$ and $n_{Cl}(z)$ respectively. From now on in all figures, black, red, blue and dark-yellow lines stand for $C_0 = 1$ M, 100mM, 10 mM and 1 mM respectively. Nanopore dimension is $L_p = 40$ nm, $R_p = 5$ nm, $\sigma_w = -49$ mC/m^2. The applied voltage $U_z = 0.1$ V.

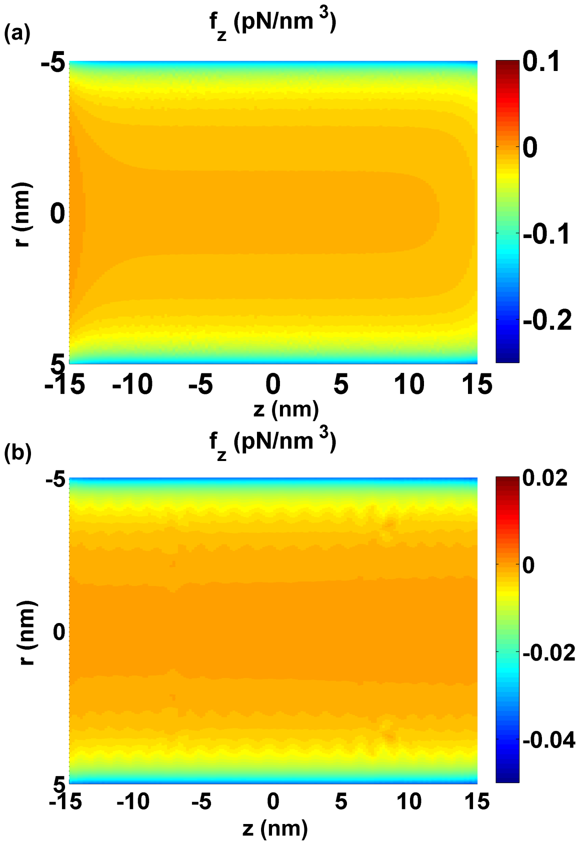

Figure S2. The 2-dimensional distribution of z-component electrical body force $f_z(r,z)$ on the solvent under salt concentration $C_0 = 1$ mM (a) and 100 mM (b), where a cross-pore pressure $\Delta p = 30$ bar is added.

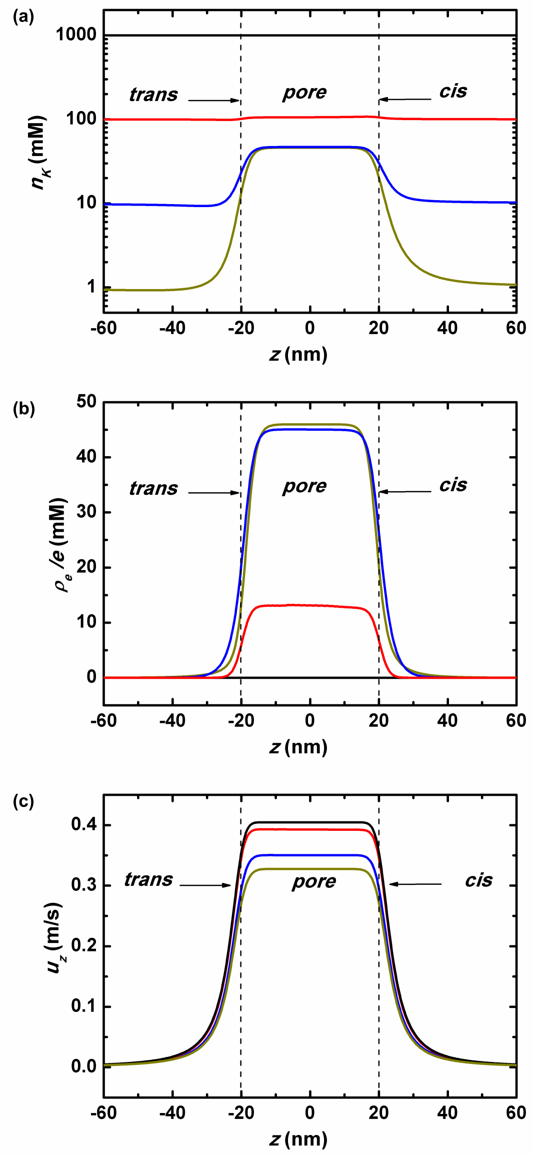

Figure S3. The pore-axial distribution of cation concentration $n_K(z)$ (a), net charge density $\rho_e/e(z)$ and z-component fluid speed $u_z(z)$ under various salt concentrations where the applied mechanical pressure $\Delta p = 30$ bar.

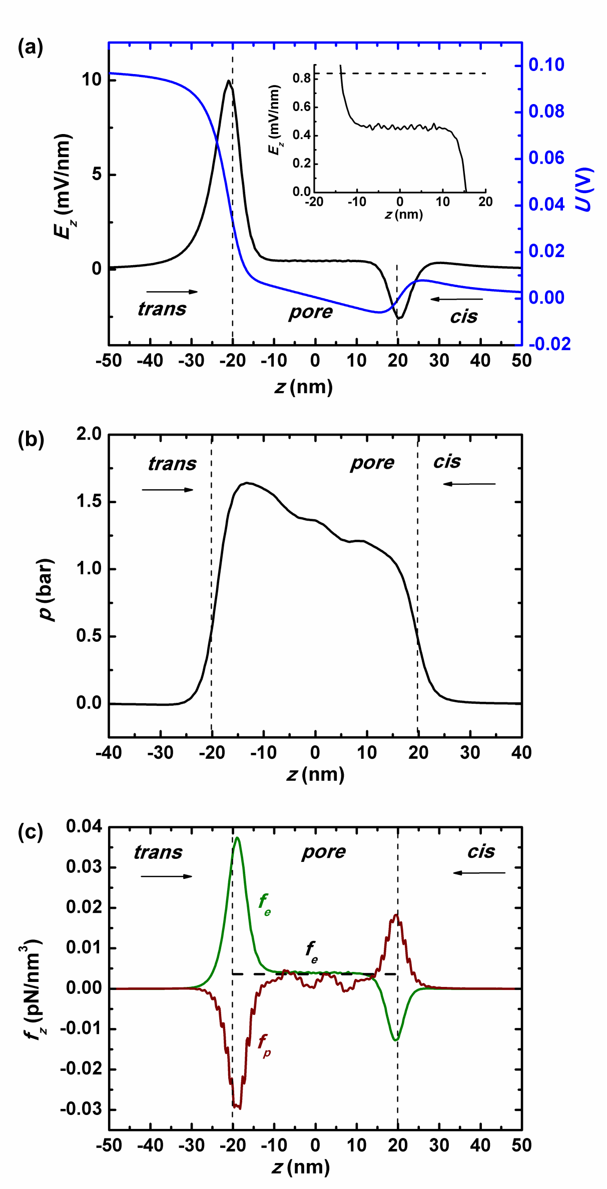

Figure S4. The distribution of voltage U and electric driving field E_z (a), pressure p (b) and z-component body force f_z (c) along the pore axis under cross-pore voltage driving. In the inset of (a), a magnified view of E_z inside the pore is provided while the dash line denote calculation result by 1-D model. Parameters: $C_0 = 10$ mM, $U = 0.1$V, $R_p = 5$ nm and $L_p = 40$ nm. In (c), the olive line characterizes the electric body force $f_e = E_z e(n_K - n_{Cl})$, the wine line is for the mechanical force $-\frac{\partial p}{\partial z}$, and the black dash line is f_e calculated by our improved 1-D model.

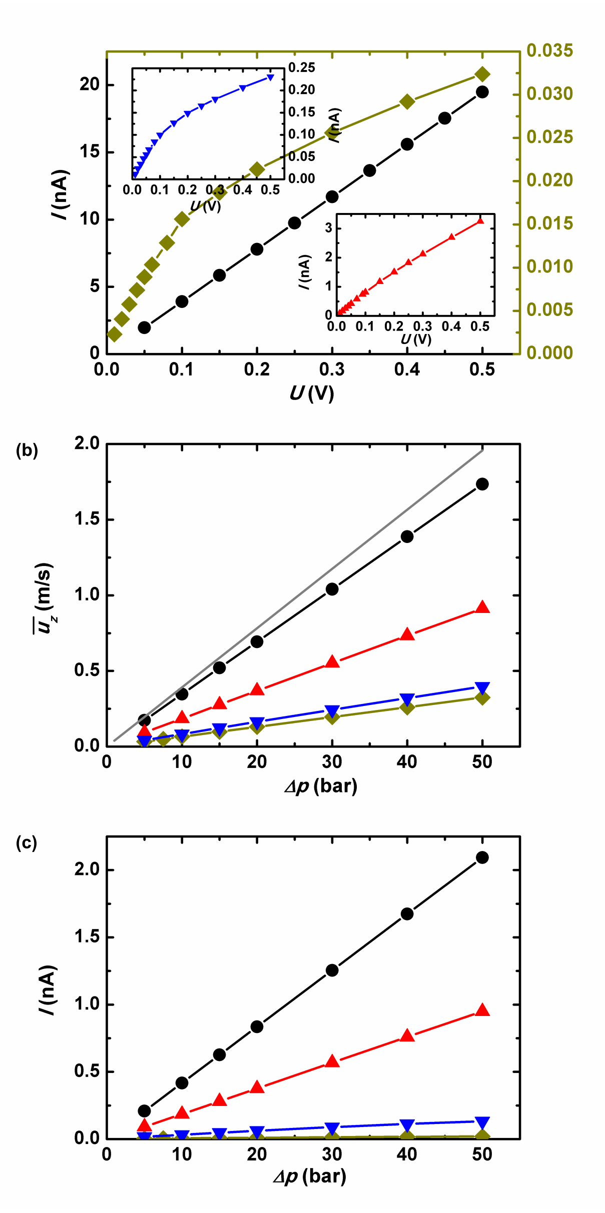

Figure S5. Transport in slippery nanopore: (a) The cross-pore ionic current I versus the applied voltage U. (b) The averaged flow velocity $\overline{u_z}$ as a function of applied hydrodynamic pressure Δp. (c) The ionic current I versus imposed cross-pore pressure Δp. The black-line-round-symbol stands for C_0 = 1 M, red-line-up-triangle for C_0 = 100 mM, blue-line-down-triangle for C_0 = 10 mM, and dark-yellow-line-rhombus-symbol for C_0 = 1 mM.

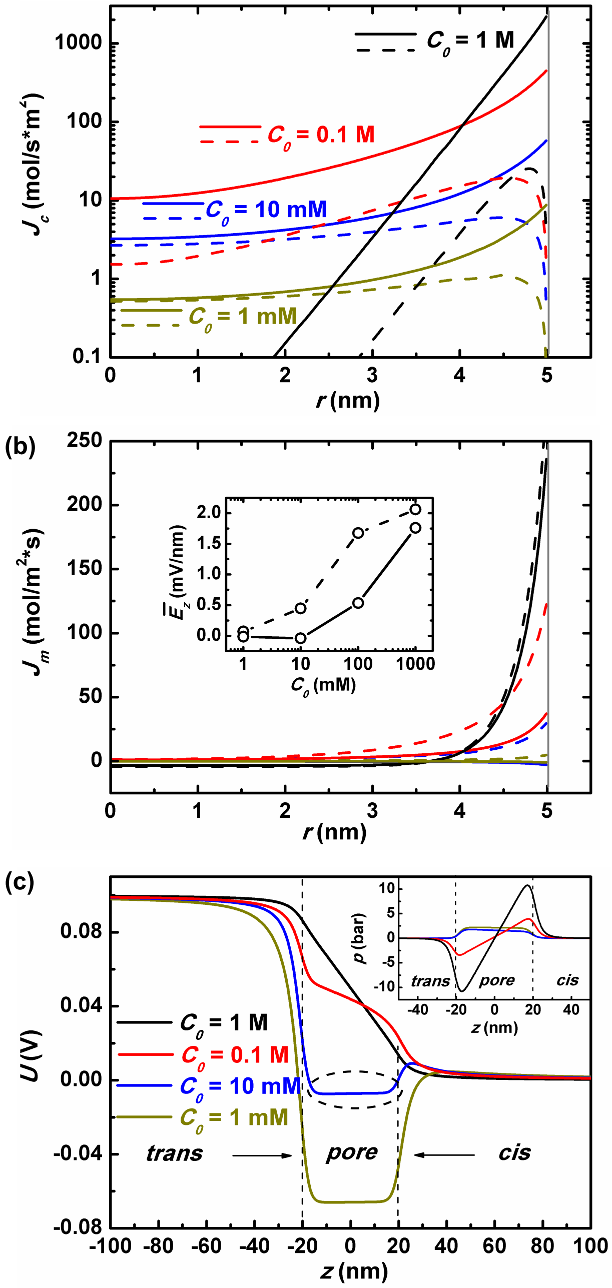

Figure S6. Electrical resistance of a slippery nanopore: The pore-radial distribution of the z-component convection flux and electrophoretic flux of ions, $J_c(r)$ (a) and $J_m(r)$ (b). The inset of (b) plots the pore-radial averaged electric driving field $\overline{E_z}$ at $z = 0$ as a function of salt concentration C_0, where the real line with symbol denotes the slip-wall situation and the dash line is for the non-slip nanopore. (c) The distribution of the voltage U and pressure p (inset) along z_0 under various salt concentrations.

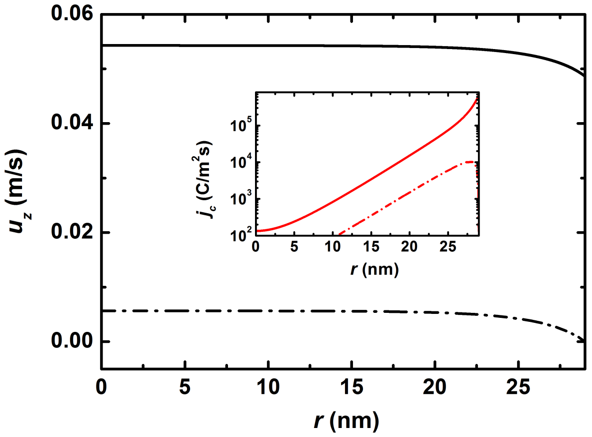

Figure S7. Fluidics in a slippery nanopore: The pore-radial distribution of z-component fluidic velocity $u_z(r)$ at the pore center ($z = 0$) in a $R_p = 30$ nm and $L_p = 900$ nm nanopore under salt concentration $C_0 = 10$ mM , mechanical pressure $\Delta p = 1$ bar (See Ref.[31] in the main context). The inset plots The pore-radial distribution of the convection ionic flux $J_c(r)$. The real lines are for the slippery nanopore while the dash lines are for the non-slip nanopore.

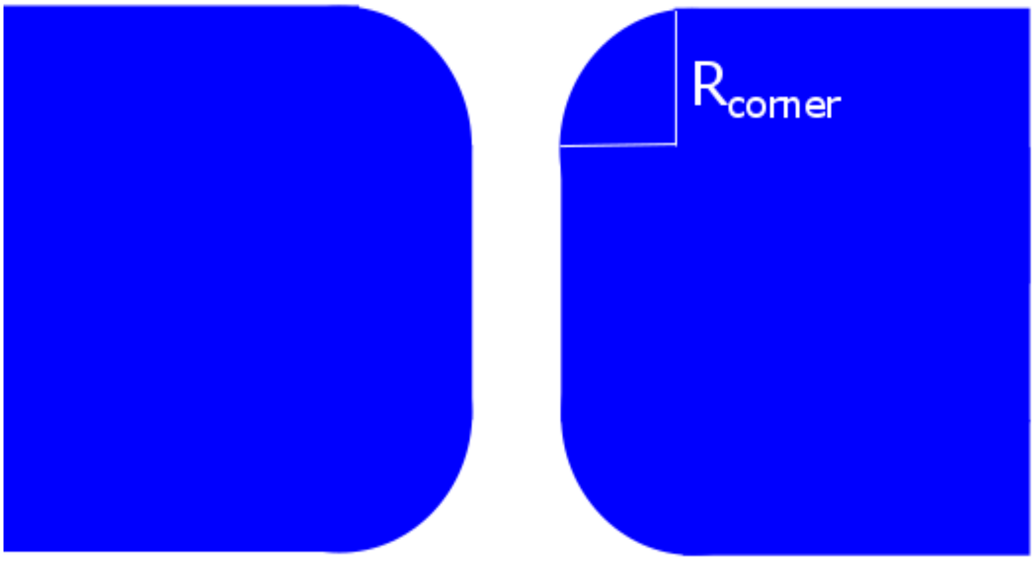

Figure S8. Schematic diagram of our model taking round corner into account (the radiu is defined as R_{corner})

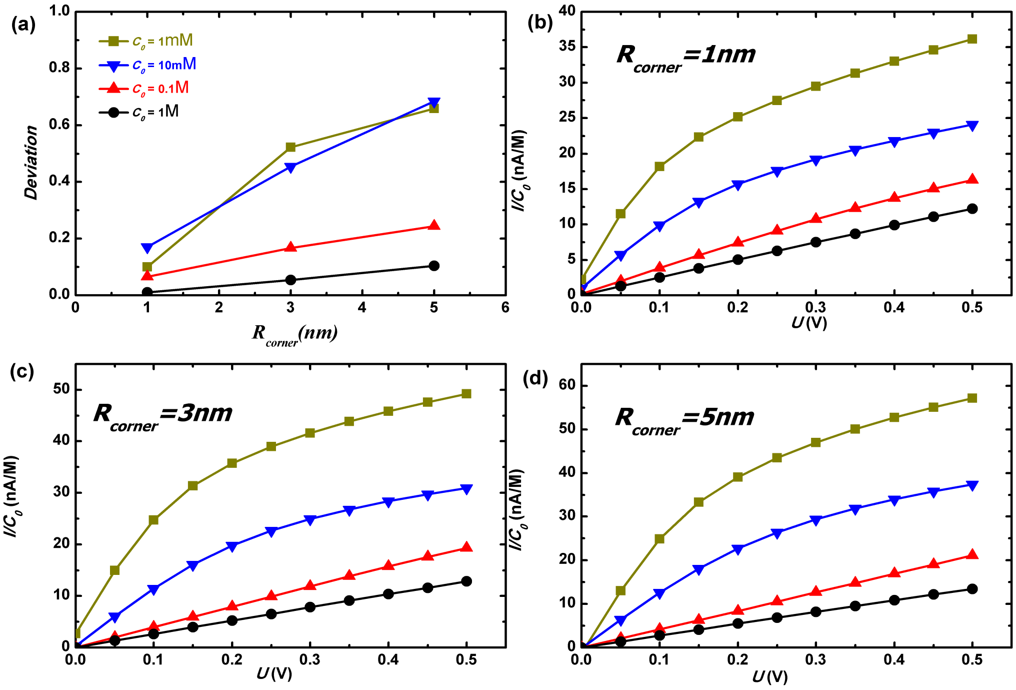

Figure S9. The influence of round edge of the nanopore on nanopore electric resistance under nonslip conditions. (a)Deviation of different R_{corner} on nanopore electrical resistance. (b),(c) and (d) are nanopore electrical resistance when R_{corner}=1,3,5nm, respectively.The other device parameters are the same as those shown in Fig.2 of the main context.

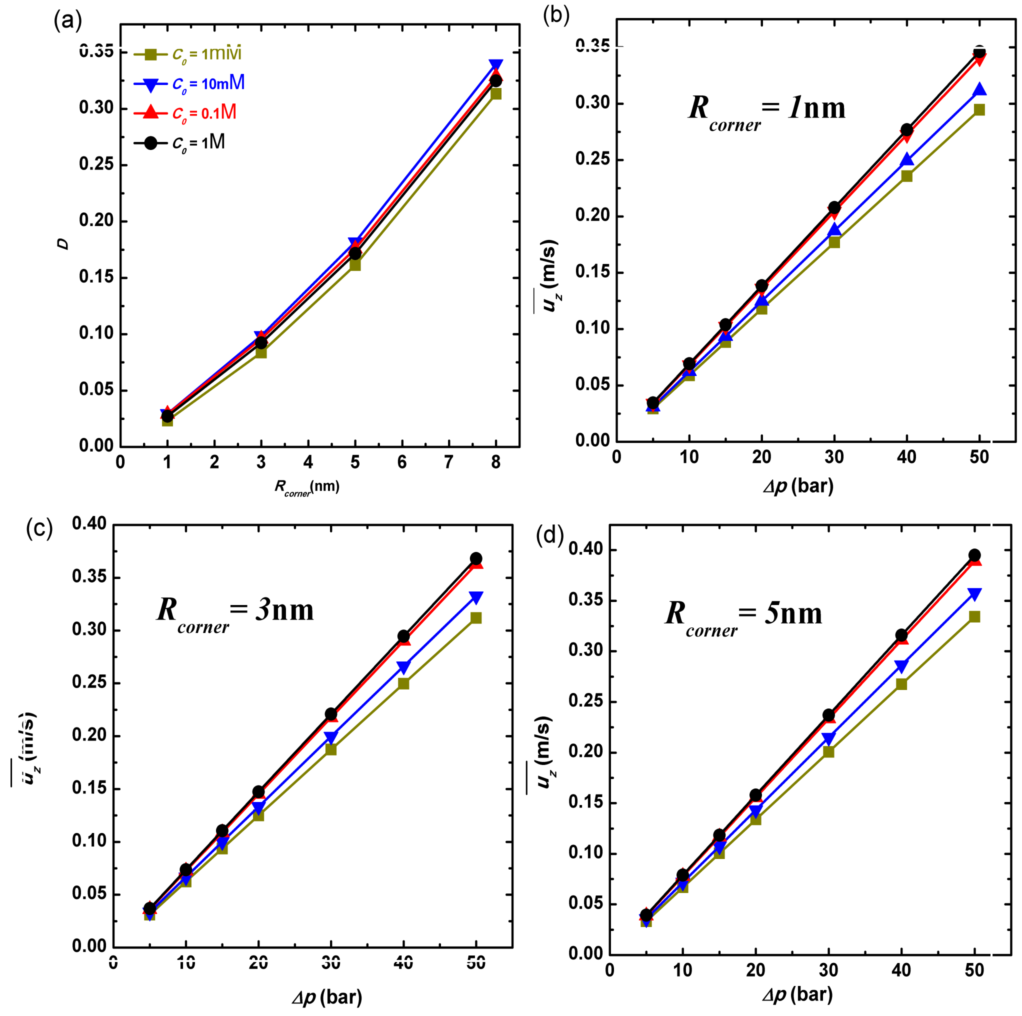

Figure S10. The influence of R_{corner} on nanopore fluidic impedence under nonslip conditions.(a)Deviation of different R_{corner} on nanopore fluidic impedance. (b),(c) and (d) are nanopore fluidic impedance when R_{corner}=1,3,5nm, respectively.The other device parameters are the same as those shown in Fig.3 of the main context.

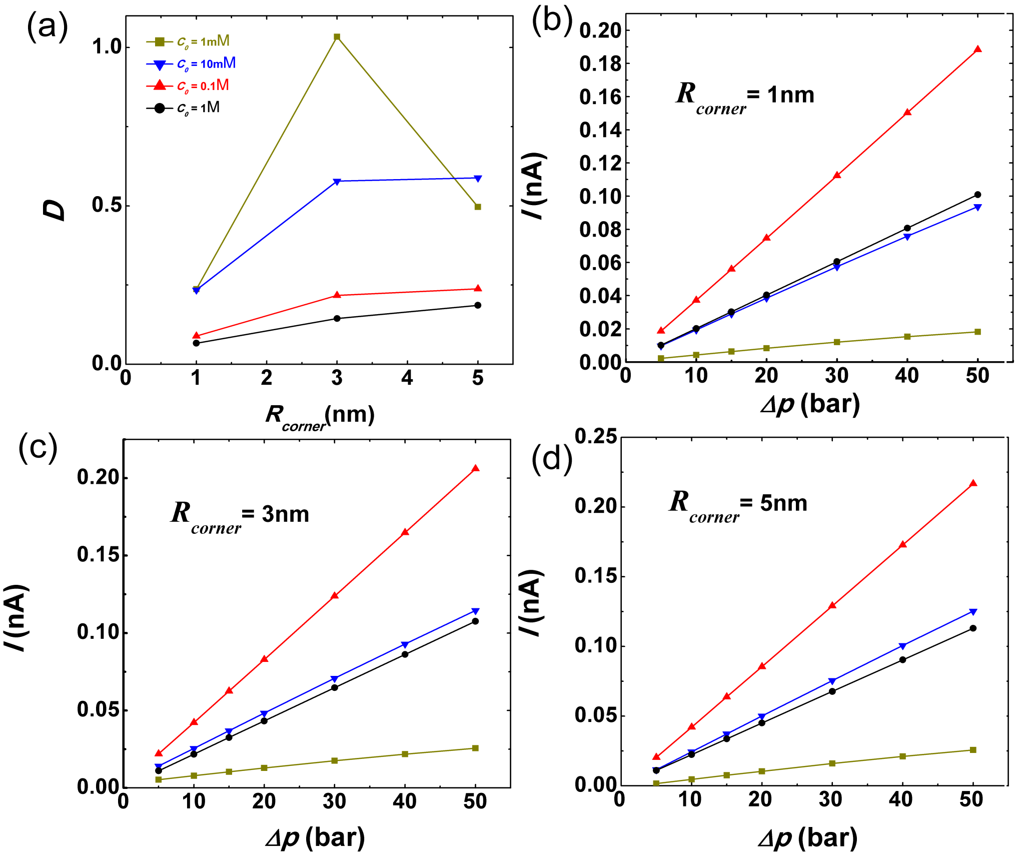

Figure S11. The influence of R_{corner} on nanopore streaming conductance S_{str} under nonslip conditions.(a)Deviation of different R_{corner} on nanopore streaming conductance S_{str} . (b),(c) and (d) are nanopore streaming conductance when R_{corner}=1,3,5nm, respectively. The other device parameters are the same as those shown in Fig.4 of the main context.

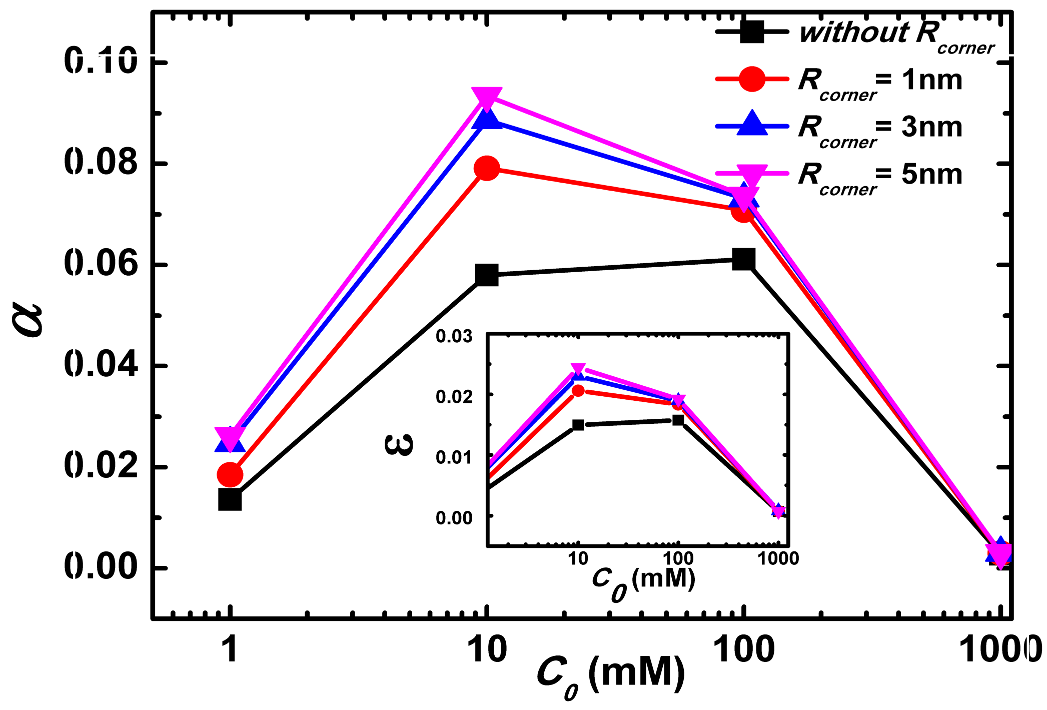

Figure S12. The influence of round edge of the nanopore on figure of merit under nonslip condition. The black, red, blue and pink lins stands for nanopore without R_{corner}, R_{corner}=1, 3, 5nm respectively.

C_0 / Model	1 mM	10 mM	0.1 M	1 M
Without σ_w	4.07×10^{10}	4.07×10^{9}	4.07×10^{8}	4.07×10^{7}
With σ_w, 1-D model	7.02×10^{9}	1.00×10^{9}	2.86×10^{8}	4.00×10^{7}
With σ_w, 2-D model	6.13×10^{9}	1.10×10^{9}	2.63×10^{8}	4.04×10^{7}

Table S1. Nanopore electrical resistance (nonslip) (Ω)

C_0 / Model	1 mM	10 mM	0.1 M	1 M
With σ_w, 2-D model R_{corner}=1nm	5.18×10^{9}	1.05×10^{9}	2.71×10^{8}	3.99×10^{7}
With σ_w, 2-D model R_{corner}=3nm	4.78×10^{9}	8.81×10^{8}	2.53×10^{8}	3.84×10^{7}
With σ_w, 2-D model R_{corner}=5nm	3.99×10^{9}	7.95×10^{8}	2.39×10^{8}	3.67×10^{7}

Table S2. The influence of round edge of the nanopore on nanopore electric resistance (nonslip) (Ω)

C_0 / Model	1 mM	10 mM	0.1 M	1 M
Without σ_w	1.89×10^{23}			
With σ_w, 1-D model	1.74×10^{23}			
With σ_w, 2-D model	2.52×10^{23}	2.31×10^{23}	1.97×10^{23}	1.89×10^{23}

Table S3. Nanopore fluidic impedance Z_{ch} (nonslip) (kg·s^{-1}·m^{-5})

Model \ C_0	1 mM	10 mM	0.1 M	1 M
With σ_w, 2-D model R_{corner}=1nm	2.16×10^{23}	2.04×10^{23}	1.87×10^{23}	1.84×10^{23}
With σ_w, 2-D model R_{corner}=3nm	2.04×10^{23}	1.91×10^{23}	1.76×10^{23}	1.73×10^{23}
With σ_w, 2-D model R_{corner}=5nm	1.91×10^{23}	1.78×10^{23}	1.64×10^{23}	1.61×10^{23}

Table S4. The influence of round edge of the nanopore on Nanopore fluidic impedance Z_{ch} (nonslip) (kg·s^{-1}·m^{-5})

Model \ C_0	1 mM	10 mM	0.1 M	1 M
Without σ_w	0			
With σ_w, 1-D model	3.53×10^{-18}	2.43×10^{-17}	4.73×10^{-17}	2.42×10^{-17}
With σ_w, 2-D model	2.97×10^{-18}	1.51×10^{-17}	3.43×10^{-17}	1.90×10^{-17}

Table S5. Nanopore streaming conductance S_{str} (nonslip) (m^3·s^{-1}·V^{-1})

Model \ C_0	1 mM	10 mM	0.1 M	1 M
With σ_w, 2-D model R_{corner}=1nm	4.06×10^{-18}	1.92×10^{-17}	3.74×10^{-17}	2.02×10^{-17}
With σ_w, 2-D model R_{corner}=3nm	5.04×10^{-18}	2.28×10^{-17}	4.06×10^{-17}	2.15×10^{-17}
With σ_w, 2-D model R_{corner}=5nm	5.87×10^{-18}	2.57×10^{-17}	4.34×10^{-17}	2.26×10^{-17}

Table S6. The influence of round edge of the nanopore on Nanopore streaming conductance S_{str} (nonslip) (m^3·s^{-1}·V^{-1})

Model \ C_0	1 mM	10 mM	0.1 M	1 M
1-D model	1.52%	10.3%	11.1%	0.408%
2-D model	1.36%	5.80%	6.11%	0.276%

Table S7. Figure of Merit (nonslip) α

Model \ C_0	1 mM	10 mM	0.1 M	1 M
2-D model R_{corner}=1nm	1.85%	7.91%	7.08%	0.299%
2-D model R_{corner}=3nm	2.48%	8.86%	7.32%	0.307%
2-D model R_{corner}=5nm	2.62%	9.34%	7.37%	0.302%

Table S8. The influence of round corner of the nanopore on the Figure of Merit (nonslip) α

Parameters \ C_0	1 mM	10 mM	0.1 M	1 M
R (Ω)	5.89×10^{9}	8.76×10^{8}	1.19×10^{8}	2.57×10^{7}
Z (kg·s^{-1}·m^{-5})	1.96×10^{23}	1.62×10^{23}	7.00×10^{22}	3.67×10^{22}
S_{str} (m^3·s^{-1}·V^{-1})	5.04×10^{-18}	3.09×10^{-17}	1.92×10^{-16}	4.19×10^{-16}
α	2.95%	13.6%	30.5%	16.6%

Table S9. Slippery nanopore parameters
